# Retrotransposons Are the Major Contributors to the Expansion of the *Drosophila ananassae* Muller F Element

**DOI:** 10.1534/g3.117.040907

**Published:** 2017-06-30

**Authors:** Wilson Leung, Christopher D. Shaffer, Elizabeth J. Chen, Thomas J. Quisenberry, Kevin Ko, John M. Braverman, Thomas C. Giarla, Nathan T. Mortimer, Laura K. Reed, Sheryl T. Smith, Srebrenka Robic, Shannon R. McCartha, Danielle R. Perry, Lindsay M. Prescod, Zenyth A. Sheppard, Ken J. Saville, Allison McClish, Emily A. Morlock, Victoria R. Sochor, Brittney Stanton, Isaac C. Veysey-White, Dennis Revie, Luis A. Jimenez, Jennifer J. Palomino, Melissa D. Patao, Shane M. Patao, Edward T. Himelblau, Jaclyn D. Campbell, Alexandra L. Hertz, Maddison F. McEvilly, Allison R. Wagner, James Youngblom, Baljit Bedi, Jeffery Bettincourt, Erin Duso, Maiye Her, William Hilton, Samantha House, Masud Karimi, Kevin Kumimoto, Rebekah Lee, Darryl Lopez, George Odisho, Ricky Prasad, Holly Lyn Robbins, Tanveer Sandhu, Tracy Selfridge, Kara Tsukashima, Hani Yosif, Nighat P. Kokan, Latia Britt, Alycia Zoellner, Eric P. Spana, Ben T. Chlebina, Insun Chong, Harrison Friedman, Danny A. Mammo, Chun L. Ng, Vinayak S. Nikam, Nicholas U. Schwartz, Thomas Q. Xu, Martin G. Burg, Spencer M. Batten, Lindsay M. Corbeill, Erica Enoch, Jesse J. Ensign, Mary E. Franks, Breanna Haiker, Judith A. Ingles, Lyndsay D. Kirkland, Joshua M. Lorenz-Guertin, Jordan Matthews, Cody M. Mittig, Nicholaus Monsma, Katherine J. Olson, Guillermo Perez-Aragon, Alen Ramic, Jordan R. Ramirez, Christopher Scheiber, Patrick A. Schneider, Devon E. Schultz, Matthew Simon, Eric Spencer, Adam C. Wernette, Maxine E. Wykle, Elizabeth Zavala-Arellano, Mitchell J. McDonald, Kristine Ostby, Peter Wendland, Justin R. DiAngelo, Alexis M. Ceasrine, Amanda H. Cox, James E.B. Docherty, Robert M. Gingras, Stephanie M. Grieb, Michael J. Pavia, Casey L. Personius, Grzegorz L. Polak, Dale L. Beach, Heaven L. Cerritos, Edward A. Horansky, Karim A. Sharif, Ryan Moran, Susan Parrish, Kirsten Bickford, Jennifer Bland, Juliana Broussard, Kerry Campbell, Katelynn E. Deibel, Richard Forka, Monika C. Lemke, Marlee B. Nelson, Catherine O'Keeffe, S. Mariel Ramey, Luke Schmidt, Paola Villegas, Christopher J. Jones, Stephanie L. Christ, Sami Mamari, Adam S. Rinaldi, Ghazal Stity, Amy T. Hark, Mark Scheuerman, S. Catherine Silver Key, Briana D. McRae, Adam S. Haberman, Sam Asinof, Harriette Carrington, Kelly Drumm, Terrance Embry, Richard McGuire, Drew Miller-Foreman, Stella Rosen, Nadia Safa, Darrin Schultz, Matt Segal, Yakov Shevin, Petros Svoronos, Tam Vuong, Gary Skuse, Don W. Paetkau, Rachael K. Bridgman, Charlotte M. Brown, Alicia R. Carroll, Francesca M. Gifford, Julie Beth Gillespie, Susan E. Herman, Krystal L. Holtcamp, Misha A. Host, Gabrielle Hussey, Danielle M. Kramer, Joan Q. Lawrence, Madeline M. Martin, Ellen N. Niemiec, Ashleigh P. O'Reilly, Olivia A. Pahl, Guadalupe Quintana, Elizabeth A.S. Rettie, Torie L. Richardson, Arianne E. Rodriguez, Mona O. Rodriguez, Laura Schiraldi, Joanna J. Smith, Kelsey F. Sugrue, Lindsey J. Suriano, Kaitlyn E. Takach, Arielle M. Vasquez, Ximena Velez, Elizabeth J. Villafuerte, Laura T. Vives, Victoria R. Zellmer, Jeanette Hauke, Charles R. Hauser, Karolyn Barker, Laurie Cannon, Perouza Parsamian, Samantha Parsons, Zachariah Wichman, Christopher W. Bazinet, Diana E. Johnson, Abubakarr Bangura, Jordan A. Black, Victoria Chevee, Sarah A. Einsteen, Sarah K. Hilton, Max Kollmer, Rahul Nadendla, Joyce Stamm, Antoinette E. Fafara-Thompson, Amber M. Gygi, Emmy E. Ogawa, Matt Van Camp, Zuzana Kocsisova, Judith L. Leatherman, Cassie M. Modahl, Michael R. Rubin, Susana S. Apiz-Saab, Suzette M. Arias-Mejias, Carlos F. Carrion-Ortiz, Patricia N. Claudio-Vazquez, Debbie M. Espada-Green, Marium Feliciano-Camacho, Karina M. Gonzalez-Bonilla, Mariela Taboas-Arroyo, Dorianmarie Vargas-Franco, Raquel Montañez-Gonzalez, Joseph Perez-Otero, Myrielis Rivera-Burgos, Francisco J. Rivera-Rosario, Heather L. Eisler, Jackie Alexander, Samatha K. Begley, Deana Gabbard, Robert J. Allen, Wint Yan Aung, William D. Barshop, Amanda Boozalis, Vanessa P. Chu, Jeremy S. Davis, Ryan N. Duggal, Robert Franklin, Katherine Gavinski, Heran Gebreyesus, Henry Z. Gong, Rachel A. Greenstein, Averill D. Guo, Casey Hanson, Kaitlin E. Homa, Simon C. Hsu, Yi Huang, Lucy Huo, Sarah Jacobs, Sasha Jia, Kyle L. Jung, Sarah Wai-Chee Kong, Matthew R. Kroll, Brandon M. Lee, Paul F. Lee, Kevin M. Levine, Amy S. Li, Chengyu Liu, Max Mian Liu, Adam P. Lousararian, Peter B. Lowery, Allyson P. Mallya, Joseph E. Marcus, Patrick C. Ng, Hien P. Nguyen, Ruchik Patel, Hashini Precht, Suchita Rastogi, Jonathan M. Sarezky, Adam Schefkind, Michael B. Schultz, Delia Shen, Tara Skorupa, Nicholas C. Spies, Gabriel Stancu, Hiu Man Vivian Tsang, Alice L. Turski, Rohit Venkat, Leah E. Waldman, Kaidi Wang, Tracy Wang, Jeffrey W. Wei, Dennis Y. Wu, David D. Xiong, Jack Yu, Karen Zhou, Gerard P. McNeil, Robert W. Fernandez, Patrick Gomez Menzies, Tingting Gu, Jeremy Buhler, Elaine R. Mardis, Sarah C.R. Elgin

**Affiliations:** a Department of Biology, Washington University in St. Louis, St. Louis, MO 63130; b Department of Biology, Saint Joseph’s University, Philadelphia, PA 19131; c Department of Biology, Siena College, Loudonville, NY 12211; d School of Biological Sciences, Illinois State University, Normal, IL 61790; e Department of Biological Sciences, University of Alabama, Tuscaloosa, AL 35401; f Department of Biology, Arcadia University, Glenside, PA 19038; g Department of Biology, Agnes Scott College, Decatur, GA 30030; h Department of Biology, Albion College, Albion, MI 49224; i Department of Biology, California Lutheran University, Thousand Oaks, CA 91360; j Department of Biological Sciences, California Polytechnic State University, San Luis Obispo, CA 93405; k Department of Biology, California State University, Stanislaus, Turlock, CA 95382; l Department of Natural Sciences, Cardinal Stritch University, Milwaukee, WI 53217; m Department of Biology, Duke University, Durham, NC 27708; n Departments of Biomedical Sciences and Cell and Molecular Biology, Grand Valley State University, Allendale, MI 49401; o Department of Biology, Hofstra University, Hempstead, NY 11549; p Department of Biological and Environmental Sciences, Longwood University, Farmville, VA 23909; q Department of Biology, Massasoit Community College, Brockton, MA 02302; r Department of Biology, McDaniel College, Westminster, MD 21157; s Department of Biological Sciences, Moravian College, Bethlehem, PA 18018; t Department of Biology, Muhlenberg College, Allentown, PA 18104; u Department of Biological & Biomedical Sciences, North Carolina Central University, Durham, NC 27707; v Department of Biology, Oberlin College, Oberlin, OH 44074; w Thomas H. Gosnell School of Life Sciences, Rochester Institute of Technology, Rochester, NY 14623; x Department of Biology, Saint Mary's College, Notre Dame, IN 46556; y Department of Biology, Simmons College, Boston, MA 02115; z Bioinformatics Program, St. Edward's University, Austin, TX 78704; aa Department of Biological Sciences, St. John's University, Queens, NY 11439; bb Department of Biological Sciences, The George Washington University, Washington, DC 20052; cc Department of Biology, University of Evansville, Evansville, IN 47722; dd Department of Biological Sciences, University of Northern Colorado, Greeley, CO 80639; ee Department of Biology, University of Puerto Rico at Cayey, Cayey, PR 00736; ff Department of Biology, University of the Cumberlands, Williamsburg, KY 40769; gg Department of Biology, York College / CUNY, Jamaica, NY 11451; hh Department of Computer Science and Engineering, Washington University in St. Louis, St. Louis, MO 63130; ii McDonnell Genome Institute, Washington University School of Medicine, St. Louis, MO 63108

**Keywords:** *Drosophila*, genome size, heterochromatin, retrotransposons, *Wolbachia*

## Abstract

The discordance between genome size and the complexity of eukaryotes can partly be attributed to differences in repeat density. The Muller F element (∼5.2 Mb) is the smallest chromosome in *Drosophila melanogaster*, but it is substantially larger (>18.7 Mb) in *D. ananassae*. To identify the major contributors to the expansion of the F element and to assess their impact, we improved the genome sequence and annotated the genes in a 1.4-Mb region of the *D. ananassae* F element, and a 1.7-Mb region from the D element for comparison. We find that transposons (particularly LTR and LINE retrotransposons) are major contributors to this expansion (78.6%), while *Wolbachia* sequences integrated into the *D. ananassae* genome are minor contributors (0.02%). Both *D. melanogaster* and *D. ananassae* F-element genes exhibit distinct characteristics compared to D-element genes (*e.g.*, larger coding spans, larger introns, more coding exons, and lower codon bias), but these differences are exaggerated in *D. ananassae*. Compared to *D. melanogaster*, the codon bias observed in *D. ananassae* F-element genes can primarily be attributed to mutational biases instead of selection. The 5′ ends of F-element genes in both species are enriched in dimethylation of lysine 4 on histone 3 (H3K4me2), while the coding spans are enriched in H3K9me2. Despite differences in repeat density and gene characteristics, *D. ananassae* F-element genes show a similar range of expression levels compared to genes in euchromatic domains. This study improves our understanding of how transposons can affect genome size and how genes can function within highly repetitive domains.

An unusual feature of eukaryotic genomes is their large variations in genome size. The size of animal genomes can range from 0.03 pg in the rice root knot nematode (*Meloidogyne graminicola*), to 3.5 pg in human, and up to 133 pg in marbled lungfish (*Protopterus aethiopicus*) (Gregory *et al.* 2007; Gregory 2016), where 1 pg corresponds to ∼978 Mb of DNA (Dolezel *et al.* 2003). The sizes of eukaryotic genomes can vary substantially even among closely related species (Bosco *et al.* 2007; Fierst *et al.* 2015). Flow cytometry analyses show that the genome sizes of 67 species within Drosophilidae range from 0.14 pg in *Drosophila mauritiana* to 0.40 pg in *Chymomyza amoena*, while maintaining a comparable number of protein-coding genes (Gregory and Johnston 2008). At broader evolutionary timescales, this lack of correlation between genome size and the genetic complexity of an organism is known as the *C*-value paradox (reviewed in Patrushev and Minkevich 2008; Eddy 2012).

One of the potential explanations for the large variations in genome sizes among different strains of the same species and between closely related species is the amount of satellite DNA and transposable elements (Bosco *et al.* 2007; Craddock *et al.* 2016; reviewed in Kidwell 2002). A previous study of 26 *Drosophila* species has shown a strong positive correlation between genome size and transposon density (Sessegolo *et al.* 2016). Because active transposons can lead to genome instability and deleterious mutations, most transposons are silenced via epigenetic and post-transcriptional silencing mechanisms (reviewed in Slotkin and Martienssen 2007; Castañeda *et al.* 2011). These silencing mechanisms could allow transposons and other repetitive sequences to persist in the genome.

DNA packaged as heterochromatin generally has higher transposon density than regions that are packaged as euchromatin (Smith *et al.* 2007). The original dichotomy between heterochromatin and euchromatin is based on the cytological staining patterns in interphase nuclei (Heitz 1928), where regions that are densely stained throughout the cell cycle are classified as heterochromatin while regions that are lightly stained in the interphase nucleus are classified as euchromatin. In addition to exhibiting higher transposon density, heterochromatic regions are late replicating, have lower gene density and lower rates of recombination, and are enriched in histone modifications such as histone 3 lysine 9 di-/trimethylation (H3K9me2/3) and chromosomal proteins such as heterochromatin protein 1a (HP1a) (reviewed in Grewal and Elgin 2007). Results from recent high-throughput chromatin immunoprecipitation (ChIP) studies of the epigenomic landscapes of metazoan genomes suggest there are multiple subtypes of heterochromatin and euchromatin (Kharchenko *et al.* 2011; Riddle *et al.* 2011; Ho *et al.* 2014).

The Muller F element in *D. melanogaster* (also known as the “dot” or the fourth chromosome in that species) is unusual in that the chromosome as a whole exhibits properties of heterochromatin (*e.g.*, late replication and low rates of recombination), but the distal portion of its long arm has a euchromatic gene density (reviewed in Riddle *et al.* 2009). The chromosome overall has an estimated size of 5.2 Mb (Locke and McDermid 1993). Its 79 genes, all located in the distal 1.3 Mb of the F element, exhibit a gene expression pattern that is similar to those of genes in euchromatin; ∼50% of the genes are active in the *D. melanogaster* S2 and BG3 cell lines across a similar range of expression levels (Riddle *et al.* 2009, 2012).

Similar to *D. melanogaster*, the F elements in other *Drosophila* species generally appear as a small “dot” chromosome (Schaeffer *et al.* 2008). Among the different *Drosophila* species, the *D. ananassae* genome is unusual; despite having only diverged from *D. melanogaster* ∼15 MYA (Obbard *et al.* 2012), the *D. ananassae* F element has undergone a substantial expansion compared to *D. melanogaster*. The estimated sizes of the *D. ananassae* and *D. melanogaster* genomes are similar [0.20 pg *vs.* 0.18 pg (Gregory and Johnston 2008)]. However, cytological studies have shown that the F element in *D. ananassae* is much larger than the small dot chromosome in *D. melanogaster*, appearing as a metacentric chromosome similar in size to the Muller A element in *D. ananassae* (Schaeffer *et al.* 2008). The *D. ananassae* F element appears to be uniformly heterochromatic in mitotic chromosome preparations (Hinton and Downs 1975), and appears as a large heterochromatic mass located near the chromocenter with a few distinct bands in polytene chromosome preparations (Kaufmann 1937). Based on the placement of the putative orthologs of *D. melanogaster* F-element genes in 16 scaffolds of the *D. ananassae* Comparative Analysis Freeze 1 (CAF1) assembly (*Drosophila* 12 Genomes Consortium 2007), Schaeffer and colleagues estimated that the *D. ananassae* F element is at least 17.8 Mb with a transposon density of 32.5% (Schaeffer *et al.* 2008).

A potential contributor to the expansion of the *D. ananassae* F element is horizontal gene transfer from the genome of the *Wolbachia* endosymbiont of *D. ananassae* (*wAna*) into the *D. ananassae* genome (reviewed in Dunning Hotopp 2011). *Wolbachia* is an intracellular bacterium that infects a large number of arthropods and nematodes (Dunning Hotopp *et al.* 2007; reviewed in Werren *et al.* 2008); a previous meta-analysis estimates that *Wolbachia* are found in ∼66% of arthropod species (Hilgenboecker *et al.* 2008). *Wolbachia* are transmitted vertically from infected females to progeny through the germline (Serbus and Sullivan 2007). To facilitate its spread, *Wolbachia* alters the reproductive system of the host to favor infected female hosts, resulting in sperm–egg cytoplasmic incompatibility, feminization of infected male hosts, and male killing (reviewed in Serbus *et al.* 2008). However, the interactions between *Wolbachia* and its hosts can also be mutualistic (Weeks *et al.* 2007; reviewed in Zug and Hammerstein 2015).

Previous studies have demonstrated that the *Wolbachia* genome is integrated into the genomes of the beetle *Callosobruchus chinensis* (Nikoh *et al.* 2008), the mosquito *Aedes aegypti* (Klasson *et al.* 2009a), and the filarial nematode *Brugia malayi* (Ioannidis *et al.* 2013). Cytological and bioinformatic analyses have indicated that the *wAna* genome is integrated into the genomes of some strains of *D. ananassae* (Klasson *et al.* 2014; Choi *et al.* 2015), including the Hawaiian strain used to construct the *D. ananassae* CAF1 assembly (*Drosophila* Species Stock Center stock number 14024-0371.13). Klasson and colleagues have previously estimated that the horizontal gene transfers and the subsequent duplications of *wAna* sequences account for ∼5 Mb (20%) of the *D. ananassae* F element (Klasson *et al.* 2014).

In this study, we performed manual sequence improvement and gene annotations of two putative *D. ananassae* F-element scaffolds. We also improved and annotated a portion of the *D. ananassae* Muller D element and used it as a reference euchromatic region in this comparative analysis. These resources have enabled us to conclude that the *D. ananassae* F element has a much higher transposon density (78.6%) than the previous estimate [32.5% (Schaeffer *et al.* 2008)]. We find that LTR and LINE retrotransposons are the major contributors to the expansion of the assembled portions of the *D. ananassae* F element, with the horizontal gene transfer of *wAna* playing only a minor role. We also examined the impact of this expansion on gene characteristics and on the epigenomic landscape of the *D. ananassae* F element in comparison to that of *D. melanogaster*. We find numerous similarities between the F elements in *D. ananassae* and *D. melanogaster*, but also detect differences in codon bias and in chromatin packaging of Polycomb-regulated genes.

## Materials and Methods

### General overview

The *D. ananassae* sequence improvement and gene annotations were organized using the framework provided by the Genomics Education Partnership (GEP) (Shaffer *et al.* 2010). Additional details on the sequence improvement and gene annotation protocols, and on the tools and tool parameters used in the bioinformatic analyses, are available in Supplemental Material, “Supplemental Methods” in File S7. The version information for the tools used in this study is available in Table S1 in File S7.

### Sequence improvement

The sequence improvement protocol and the quality standards for the improved regions have been described previously (Slawson *et al.* 2006; Leung *et al.* 2010). The *D. ananassae* CAF1 assembly (*Drosophila* 12 Genomes Consortium 2007) was obtained from the AAA: 12 *Drosophila* Genomes Web site (http://eisenlab.org/AAA/index.html). The fosmid clones for the *D. ananassae* analysis regions improved_13010, improved_13034_2, and improved_13337 were obtained from the *Drosophila* Genomics Resource Center at Indiana University. These fosmid clones were used as templates for additional sequencing reactions and to produce the restriction digests for verifying the final assembly. Additional sequencing data for the improved_13034_1 region was produced by sequencing genomic PCR products. The *D. ananassae* genomic DNA (derived from the same strain used in the original sequencing project) was obtained from the *Drosophila* Species Stock Center at the University of California, San Diego (stock number 0000-1005.01).

The final assemblies for the improved_13010, improved_13034_2, and improved_13337 regions were confirmed by multiple restriction digests of the overlapping fosmids (Figure S2A in File S7). The improved_13034_1 assembly was confirmed by subreads produced by the Pacific Biosciences (PacBio) RS II sequencer (Figure S2B in File S7). In collaboration with the McDonnell Genome Institute, the Single Molecule, Real Time (SMRT) sequencing of the *D. ananassae* genome was performed using eight SMRT cells with the DNA Template Prep Kit 2.0 (3–10 kb), the DNA/Polymerase Binding Kit 2.0 (24 Rxn), the C2 chemistry, and a movie duration of 120 min.

### Gene annotations

The gene annotation protocol has been described previously (Shaffer *et al.* 2010; Leung *et al.* 2015). For quality control purposes, each annotation project was completed by two or more GEP students working independently. Experienced students working under the supervision of GEP staff used *Apollo* (Lewis *et al.* 2002) to reconcile these gene annotations, and to create the gene models analyzed in this study. The annotations of the *D. ananassae* genes used the release 6.06 *D. melanogaster* gene annotations produced by FlyBase as a reference (Attrill *et al.* 2016). Release 1.04 of the *D. ananassae* gene predictions were obtained from FlyBase, and the UCSC *liftOver* utility was used to migrate these predictions to the improved *D. ananassae* assembly. Seven gene predictions (FBtr0115589, FBtr0115686, FBtr0128102, FBtr0128153, FBtr0381268, FBtr0384375, and FBtr0385219) could not be transferred to the improved assembly because of changes to the genomic sequences as a result of sequence improvement.

### Repeat density analysis

*Repeat Detector* (*Red*) (Girgis 2015) and *WindowMasker* (Morgulis *et al.* 2006) were run against the improved *D. ananassae* assembly using default parameters. For the *Tallymer* analysis (Kurtz *et al.* 2008), the word sizes (k) of 17 and 19 were used to analyze the *D. melanogaster* and the *D. ananassae* assemblies, respectively (see “Supplemental Methods” in File S7 for the procedures used to select these word sizes). Simple and low complexity repeats were identified using *tantan* (Frith 2011) with the default masking rate (−r 0.005). Tandem repeats were identified using *Tandem Repeats Finder* (*TRF*) (Benson 1999) with the parameters: Match = 2, Mismatch = 7, Delta = 7, Match Probability = 80, Mismatch Probability = 10, Minscore = 50, and MaxPeriod = 2000.

Transposon remnants were identified using *RepeatMasker* (Smit* et al.* 2013) with the RepBase library [release 20150807 (Jurka *et al.* 2005)]. *RepeatMasker* was run on the *D. melanogaster* and *D. ananassae* assemblies using the WU BLAST search engine (-e wublast) at the most sensitive setting (-s) against *Drosophila* sequences in the RepBase library (-species drosophila), without masking low complexity and simple repeats (-nolow). Overlapping repeats were merged into a single interval if they belonged to the same class of transposon, and reclassified as the “overlapping” class if they belonged to difference classes of transposons.

### Estimating the density of Wolbachia fragments

The assemblies for the *Wolbachia* endosymbionts of *D. ananassae* [*wAna* (Salzberg *et al.* 2005)], *D. simulans* strain Riverside [*wRi* (Klasson *et al.* 2009b)], and *D. melanogaster* [*wMel* (Wu *et al.* 2004)] were obtained from the NCBI BioProject database using the accession numbers PRJNA13365, PRJNA33273, and PRJNA272, respectively. Each *Wolbachia* assembly was compared against the *D. melanogaster* and the improved *D. ananassae* assemblies using *RepeatMasker* with the following parameters: -e wublast -s -nolow. The *RepeatMasker* results against the three *Wolbachia* assemblies were filtered using cutoff scores (255.6–263.4 for *D. melanogaster* and 250.7–256.0 for *D. ananassae*) to reduce spurious matches.

The *Wolbachia* protein sequences for *wAna*, *wRi*, and *wMel* were obtained from the NCBI Protein database using the BioProject accession numbers PRJNA13365, PRJNA33273, and PRJNA272, respectively. These protein sequences were compared against the *D. melanogaster* and *D. ananassae* assemblies using *CENSOR* (Kohany *et al.* 2006) with the WU BLAST (Gish 1996) *tblastn* module (-bprg tblastn) at the sensitive (-s) setting. The results were filtered by cutoff scores (112.0–116.2 for *D. melanogaster* and 94.8–106.2 for *D. ananassae*) to reduce the number of spurious matches (see “Supplemental Methods” in File S7 for the protocol used to determine the cutoff scores for the *RepeatMasker* and *CENSOR* searches).

### Analysis of the wAna assembly

The *wAna* assembly was aligned against the *wRi* and *wMel* assemblies using *LAST* (Kiełbasa *et al.* 2011) with default parameters. These alignments were filtered and chained together using the UCSC Chain and Net alignment protocol (Kent *et al.* 2003). The regions of the *wAna* assembly that aligned to the improved *D. ananassae* assembly were extracted from the *RepeatMasker* results produced as part of the *Estimating the density of Wolbachia fragments* analysis. Regions within the *wAna* assembly that showed sequence similarity to *Drosophila* transposons in the *RepBase* library [release 20150807 (Jurka *et al.* 2005)] were identified using *RepeatMasker* (Smit *et al.* 2013) with the parameters: -s -e wublast -nolow -species drosophila.

### Gene characteristics analysis

The isoform with the largest total coding exon size (*i.e.*, the most comprehensive isoform) was used in the analysis of gene characteristics. Because the analysis focused only on the coding regions, the total intron size metric for *D. ananassae* and *D. melanogaster* only included the introns that were located between coding exons. Similarly, only the internal coding exons and the translated portions of the first and last coding exons were included in the analysis of coding exon sizes, even though the transcribed exons might be larger because of untranslated regions.

The statistical analyses of gene characteristics were performed using *R* (R Core Team 2015). Violin plots were created using a modified version of the *vioplot* function in the *R* package vioplot (Adler 2005). Modifications to the *vioplot* function were made to highlight the interquartile range (IQR) in each violin plot. For violin plots using the log_10_ scale, a pseudocount of 1 was added to all the values.

The Kruskal–Wallis rank sum test (KW test) (Kruskal and Wallis 1952) (the *kruskal.test* function in R) was used to determine if the differences in gene characteristics among the four analysis regions are statistically significant [type I error (α) = 0.05]. Following the rejection of the null hypothesis by the KW test, *post hoc* Dunn tests (DT) (Dunn 1964) were performed using the *dunn.test* function in the *R* package dunn.test (Dinno 2016) to identify the pairs of analysis regions that show significantly different distributions. The Holm–Bonferroni method (method=“holm”) was used by the DT to control the family-wise error rate (Holm 1979). Rejection of the null hypothesis using the Holm–Bonferroni method depends on both the adjusted p-values (adjp) and the order of the unadjusted p-values.

### Codon bias analysis

The codon bias analysis protocol has previously been described (Leung *et al.* 2015). The effective number of codons (Nc) and the codon adaptation index (CAI) for each gene were determined by the *chips* and *cai* programs in the *EMBOSS* package (Rice *et al.* 2000), respectively (see “Supplemental Methods” in File S7 for the procedure used to construct the reference gene set for the CAI analysis). The violin plots and the KW tests were performed using the procedure described in the *Gene characteristics analysis* section. The codon frequency and the codon GC content for the genes in the *D. melanogaster* and *D. ananassae* analysis regions were determined by the *cusp* program in the *EMBOSS* package.

Locally estimated scatterplot smoothing (LOESS) (Cleveland and Devlin 1988) was applied to each Nc *vs.* CAI scatterplot to delineate the major trends. The span parameter for the LOESS regression model was determined by generalized cross-validation using the *loess.as* function in the *R* package fANCOVA with the parameters: degree = 1, criterion=“gcv,” family=“symmetric.” The scatterplot and the LOESS curve were created using the *scatter.smooth* function in *R*.

### Melting temperature metagene profile

The protocol for creating the melting temperature (T_m_) metagene profile has previously been described (Leung *et al.* 2015). The T_m_ in the *D. melanogaster* and the *D. ananassae* analysis regions were determined using the *dan* program in the *EMBOSS* package with a 9-bp sliding window (-windowsize = 9), a step size of one (-shiftincrement = 1), and the following parameters: -dnaconc = 50 -saltconc = 50 -mintemp = 55. The T_m_ for the coding span was standardized to 3 kb. The metagene consisted of the standardized 3-kb coding span and the 2-kb region upstream and downstream of the coding span. The median T_m_ at each position of the metagene was calculated, and a cubic smoothing spline was fitted to the median T_m_ profile using the *smooth.spline* function in *R* with ordinary cross-validation (cv = TRUE).

### Chromatin immunoprecipitation sequencing analysis

Detailed descriptions of the chromatin immunoprecipitation sequencing analysis (ChIP-Seq) read mapping and peak calling protocols are available in “Supplemental Methods” in File S7. The *D. ananassae* ChIP-Seq data were produced using samples from the third instar larval stage of development. The chromatin isolation and immunoprecipitation protocols have previously been described (Riddle *et al.* 2012). The antibodies (Abcam ab1220 for H3K9me2, Millipore 07-030 for H3K4me2, and Abcam ab6002 for H3K27me3) have previously been validated by the modENCODE project (Egelhofer *et al.* 2011). Eight samples (two biological replicates of the ChIP for the three histone modifications and input DNA) were sequenced by the Genome Technology Access Center at Washington University in St. Louis using a single lane on the Illumina HiSeq 2000 sequencer, producing paired-end reads with read lengths of 101 bp. The ChIP-Seq reads were mapped against the improved *D. ananassae* assembly using *BWA-MEM* (Li 2013) with default parameters.

The third instar larvae ChIP-Seq data for the Oregon-R strain of *D. melanogaster* were produced by the modENCODE project (Landt *et al.* 2012; Ho *et al.* 2014). These ChIP-Seq data and input DNA controls were obtained from the European Nucleotide Archive (ENA) using the accession numbers SRP023384 (H3K9me2), SRP023385 (H3K4me2), and SRP028497 (H3K27me3). The ChIP-Seq samples were sequenced using the Illumina Genome Analyzer, producing unpaired reads with a read length of 50 bp. The ChIP-Seq reads were mapped against the *D. melanogaster* assembly using *BWA-backtrack* (Li and Durbin 2009) with default parameters.

Regions enriched in these three histone modifications were identified using *MACS2* (Zhang *et al.* 2008). The log-likelihood-enrichment ratios (LLR) of the ChIP samples compared to DNA input controls were calculated by the *bdgcmp* subprogram in *MACS2* using the following parameters: -m logLR -p 0.00001. The LLR metagene profiles were created using the technique described in the *Melting temperature metagene profile* section.

### RNA-Seq expression analysis

In addition to the *D. ananassae* adult males and adult females RNA-Seq samples [SRP006203 (Graveley *et al.* 2011)] used in gene annotations, RNA-Seq data from other developmental stages and tissues were obtained from the ENA. The 76-bp, paired-end RNA-Seq data produced using the Illumina HiSeq 2500 sequencer for *D. ananassae* virgin female carcass, male carcass, female ovaries, and male testes were obtained from the ENA using the accession number SRP058321 (Rogers *et al.* 2014). The 75-bp, paired-end RNA-Seq data produced by the Illumina Genome Analyzer IIx sequencer from *Wolbachia*-cured *D. ananassae* embryos were obtained from the ENA using the accession number SRP007906 (Kumar *et al.* 2012).

The RNA-Seq reads from these seven samples were mapped against the improved *D. ananassae* assembly using two rounds of *HISAT2* (Kim *et al.* 2015). The RNA-Seq read counts for each gene were determined by *htseq-count* (Anders *et al.* 2015) using default parameters. The *D. ananassae* gene predictions with no mapped reads in any of the seven RNA-Seq samples were omitted from the analysis. The regularized log (rlog) transformed expression level of each *D. ananassae* gene was calculated by the *rlogTransformation* function in *DESeq2*, where the experimental design information was used in the calculation of the gene-wise dispersion estimates (blind = FALSE). The distributions of rlog values for all *D. ananassae* genes, genes on the F element, and genes at the base of the D element were analyzed using violin plots and KW tests with the protocols described in the *Gene characteristics analysis* section.

### Data availability

The *D. ananassae* ChIP-Seq data are available at the NCBI BioProject database with the accession number PRJNA369805. The *D. ananassae* PacBio whole-genome sequencing data are available at the NCBI BioProject database with the accession number PRJNA381646. The improved *D. ananassae* sequences and gene annotations, and the results of the bioinformatic analyses for *D. ananassae* and *D. melanogaster*, are available through the GEP UCSC Genome Browser Mirror (http://gander.wustl.edu).

## Results

### Sequence improvement of the D. ananassae F-element assembly

The *D. ananassae* CAF1 assembly was constructed by the *Drosophila* 12 Genomes Consortium through the reconciliation of the whole-genome shotgun (WGS) assemblies from multiple assemblers (*Drosophila* 12 Genomes Consortium 2007; Zimin *et al.* 2008). Earlier studies have shown that the F element has a higher repeat density compared to the other autosomes (reviewed in Riddle *et al.* 2009), which would likely result in a higher frequency of misassemblies (Salzberg and Yorke 2005). In this study, we performed manual sequence improvement of three regions from the *D. ananassae* F element (improved_13010, improved_13034_1, and improved_13034_2) and a euchromatic reference region near the base of the D element (improved_13337). These regions are labeled as “*D. ana*: F (improved)” and “*D. ana*: D (improved)” in the subsequent analysis, respectively (see Table S2 in File S7 for the coordinates of the improved regions and the corresponding comparison regions in *D. melanogaster*).

These sequence improvement efforts produce a 1.4-Mb, high-quality region for the *D. ananassae* F element (Table 1A) and a 1.7-Mb, high-quality region for the D element (Table 1B). The manual sequence improvement closed 35 gaps in the CAF1 assembly, adding a total of 26,266 bases. Net alignments between the improved regions and the corresponding regions in the CAF1 assembly show a total of 249 differences, with a total size difference of 79,735 bases. The large size differences could primarily be attributed to collapsed repeats in the CAF1 assembly. Some of the highly repetitive regions within the improved regions remained unresolved (6% in the F element and 2% in the D element). See the distributions of the problem regions in Figure S1 in File S7.

**Table 1:** Sequence improvement statistics for the *D. ananassae* F- and D-element regions studied

Region	Total (bp)	Gap (bp)	Unresolved (bp)	Low Quality (bp)
**A**				
improved_13010	597,760	495	7048	16
improved_13034_1	490,783	50	70,695	944
improved_13034_2	395,316	25	7204	392
Total	1,483,859	570	84,947	1352
**B**				
improved_13337	1,708,805	0	30,512	709

Unresolved regions generally correspond to areas with large tandem or inverted repeats. Regions with *Phred* scores <30 (*i.e.*, estimated error rate >1/1000 bases) are classified as low quality. Most low quality regions either overlap with unresolved regions or are located adjacent to gaps. (A) One region from the F-element scaffold improved_13010 and two regions from the F-element scaffold improved_13034 were improved, which results in a total of 1.4 Mb of high quality bases. (B) The region near the base of the D element (scaffold improved_13337) was improved, which results in 1.7 Mb high quality bases.

**Figure 1:**
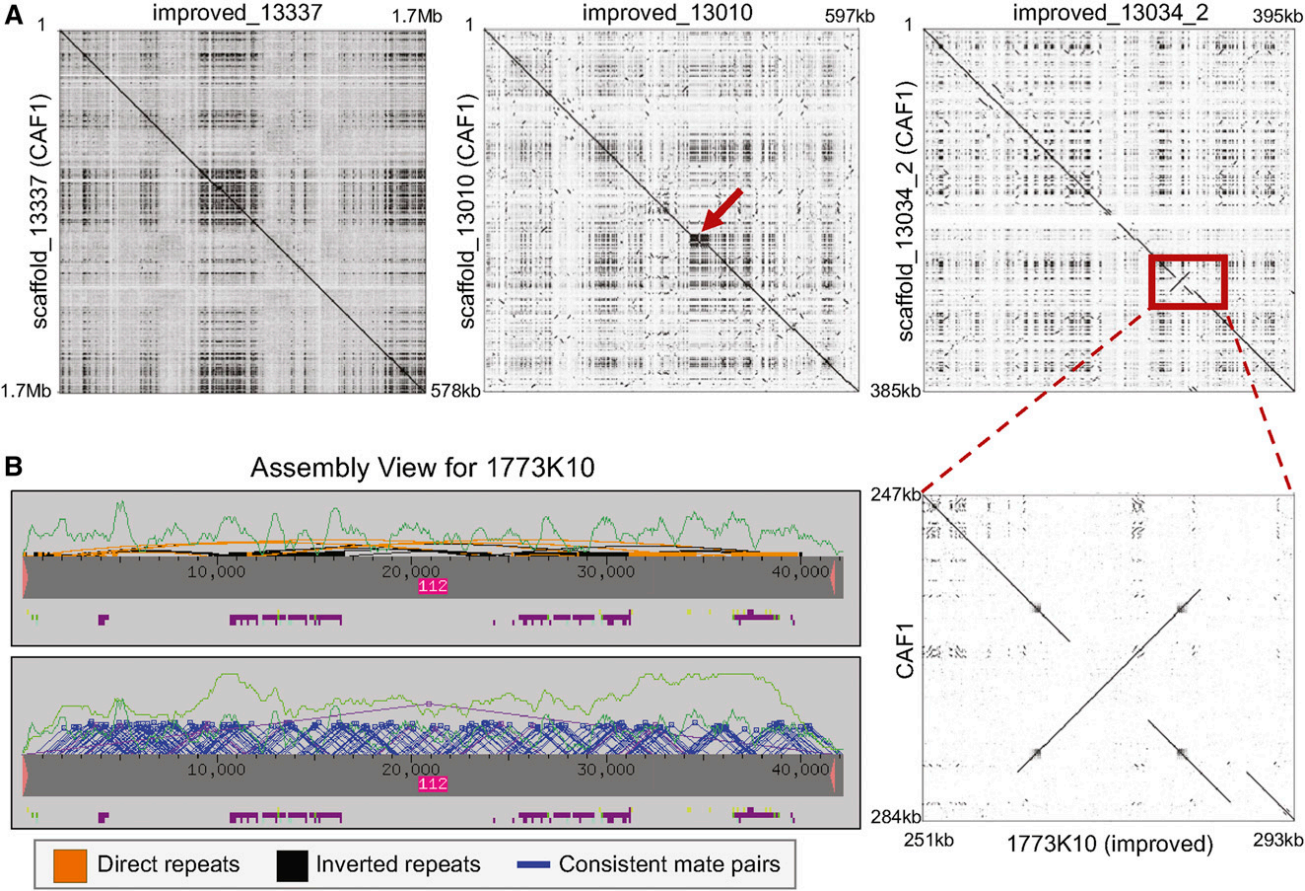
Results of the manual sequence improvement of the *D. ananassae* D and F elements. (A) Dot plot comparisons of the scaffolds in the original CAF1 assembly (*y*-axis) *vs.* the scaffolds in the improved assembly (*x*-axis). Dots within each dot plot denote regions of similarity between the CAF1 assembly and the improved assembly. The diagonal lines in the dot plots for the D-element scaffold improved_13337 (left) and the F-element scaffold improved_13010 (middle) show that the overall CAF1 assemblies for these regions are consistent with the corresponding assemblies following manual sequence improvement. However, the high density of dots in the middle of the dot plot for improved_13010 corresponds to a collapsed repeat within the CAF1 assembly (red ←). Manual sequence improvement also identified a major misassembly in the second improved region (improved_13034_2) within the F-element scaffold_13034 (right), where part of the scaffold was inverted compared to the final assembly (red box). The misassembled region is part of the fosmid 1773K10 (bottom inset). (B) The *Consed* Assembly View for the improved fosmid project 1773K10 shows that the misassembled region contains multiple tandem and inverted repeats. (Top) The gray bar within the Assembly View corresponds to the improved fosmid assembly, and the pink Δ’s denote the ends of the fosmid. The purple and green boxes underneath the gray box correspond to tags (*e.g.*, repeats and comments), and the dark green line corresponds to the read depth. The orange and black boxes above the gray bar correspond to tandem and inverted repeats, respectively. These orange and black boxes indicate that the improved assembly contains multiple tandem and inverted repeats that are located adjacent to each other. (Bottom) The improved assembly for this fosmid was supported by consistent forward-reverse mate pairs (blue Δ’s) and by multiple restriction digests (Figure S2A in File S7).

Dot plot alignments show that the improved scaffolds and the corresponding scaffolds in the CAF1 assembly are generally consistent with each other (Figure 1A, left and center). However, there is a major misassembly (inversion) in the second improved region within the scaffold improved_13034 (red box in Figure 1A right). This region is covered by the fosmid 1773K10 and manual sequence improvement revealed that the misassembled region contains a tandem repeat that is located adjacent to an inverted repeat (Figure 1B). The final assembly for the fosmid 1773K10 is supported by consistent forward-reverse mate pairs (Figure 1B, bottom) and the *Eco*RI and *Hind*III restriction digests (Figure S2A in File S7).

As part of the sequence improvement standards used in this study, the fosmid projects in the improved_13337, improved_13010, and improved_13034_2 regions are all supported by at least two restriction digests. The projects in the improved_13034_1 region are supported by long overlapping subreads produced by the PacBio sequencer (Figure S2B in File S7). In conjunction with the consistent forward-reverse mate pairs, these resources provide external confirmation of the final assembly and provide more accurate gap size estimates.

In addition to improving the assemblies, we also created a set of manual gene annotations for these improved regions, using the gene models in *D. melanogaster* as a reference. Collectively, we annotated 13 genes on the improved *D. ananassae* F-element regions and 125 genes at the base of the D element (see File S1 for the list of noncanonical features and differences in gene or isoform structures compared to the *D. melanogaster* orthologs).

Because of the low levels of sequence similarity between the untranslated regions of *D. melanogaster* genes and the *D. ananassae* assembly, we only annotated the coding regions of the *D. ananassae* genes and focused our analyses on the properties of the coding span (*i.e.*, the region from start codon to stop codon, including introns). To avoid counting the same genomic region multiple times due to alternative splicing, we also restricted our analyses to the most comprehensive isoform for each gene (*i.e.*, the isoform with the largest total coding exon size).

### Identifying D. ananassae F element scaffolds

Earlier work has demonstrated that ∼95% of the *D. melanogaster* genes remain on the same Muller element across 12 *Drosophila* species that diverged >40 MYA (Bhutkar *et al.* 2008). To increase the statistical power of the comparative analysis with *D. melanogaster*, we identified additional *D. ananassae* F-element scaffolds based on sequence similarity to protein-coding genes on the *D. melanogaster* F element.

This analysis identified 64 complete *D. ananassae* F-element genes on 21 scaffolds [labeled “*D. ana*: F (all)” in the following analysis], with a total size of 18.7 Mb (see “Supplemental Methods” in File S7 and File S2 for details). These *D. ananassae* gene annotations are available through the GEP UCSC Genome Browser Mirror at http://gander.wustl.edu/ [*D. ananassae* (GEP/DanaImproved) assembly]. Using these resources and the orthologous regions from *D. melanogaster* [labeled “*D. mel*: F” and “*D. mel*: D (base)” in the following analysis, respectively], we performed a comparative analysis to examine the factors that contribute to the expansion of the *D. ananassae* F element and the impact of this expansion on gene characteristics.

### Retrotransposons are the major contributors to the expansion of the D. ananassae F element

**Figure 2:**
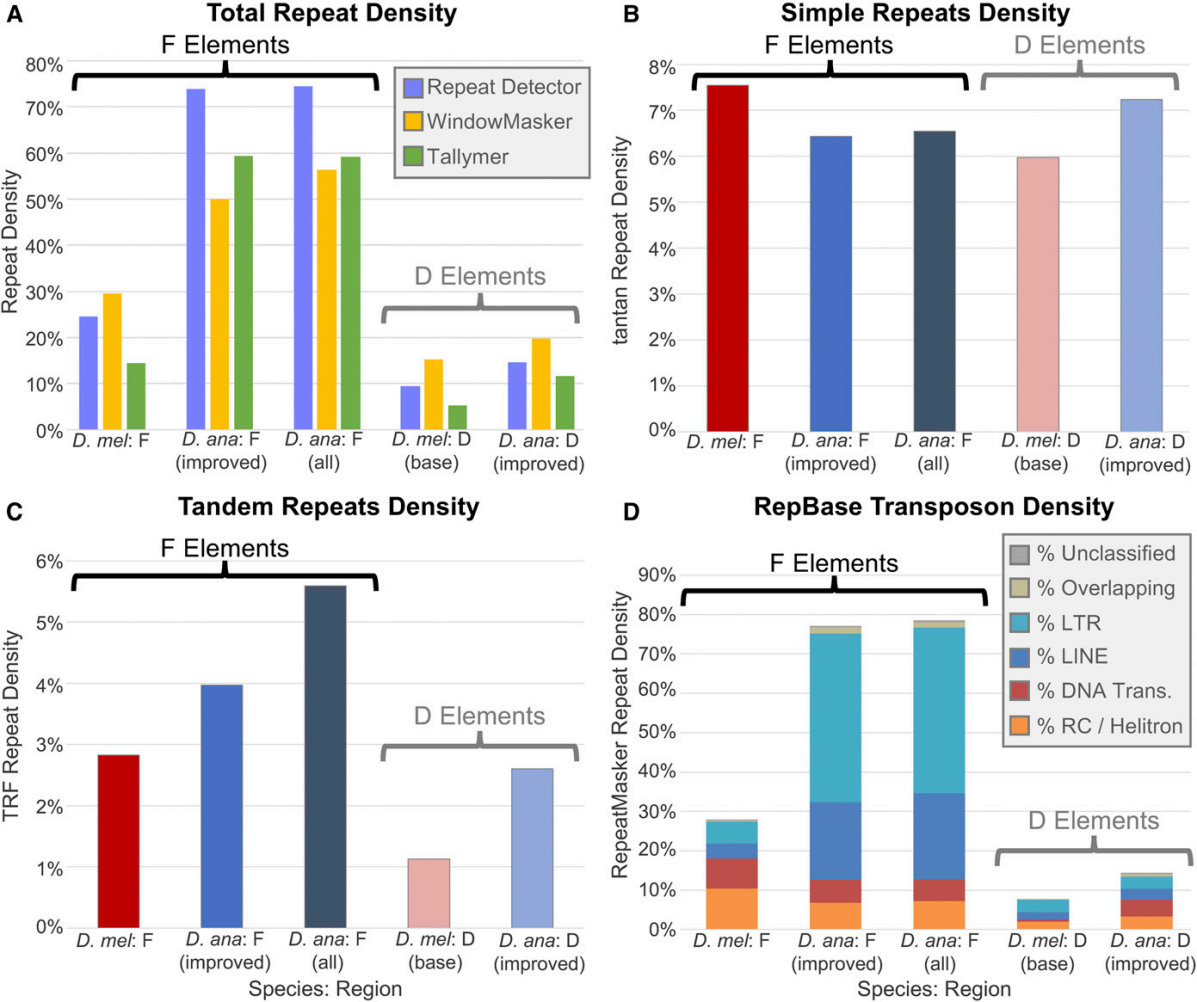
Expansion of the *D. ananassae* F element can primarily be attributed to the high density of LTR and LINE retrotransposons. (A) Total repeat density estimates from *de novo* repeat finders (*Red*, *WindowMasker*, and *Tallymer*) show that the *D. ananassae* F element has higher repeat density than the *D. melanogaster* F element (56.4–74.5% *vs.* 14.4–29.5%). Both F elements show higher repeat density than the euchromatic reference regions from the base of the D elements in *D. ananassae* (11.6–19.8%) and in *D. melanogaster* (5.4–15.2%). The repeat densities of the improved *D. ananassae* F-element scaffolds [*D. ana*: F (improved)] are similar to the repeat densities for all *D. ananassae* F-element scaffolds [*D. ana*: F (all)]. (B) Results from the *tantan* analysis show that the five analysis regions from *D. melanogaster* and *D. ananassae* have similar simple repeat density (6.0–7.5%). (C) *TRF* analysis shows that *D. ananassae* has higher tandem repeats density than *D. melanogaster* on both the F (5.6 *vs.* 2.8%) and the D elements (2.6 *vs.* 1.1%). (D) *RepeatMasker* analysis using the *Drosophila* RepBase library shows that the F element has higher transposon density than the D element both in *D. melanogaster* (28.0 *vs.* 7.7%) and in *D. ananassae* (78.6 *vs.* 14.4%). There is a substantial increase in the density of LTR and LINE retrotransposons on the *D. ananassae* F element compared to the *D. melanogaster* F element (42.1 *vs.* 5.5% and 21.8 *vs.* 3.8%, respectively). The *D. ananassae* euchromatic reference region also shows higher transposon density than *D. melanogaster*, but most of the difference can be attributed to the density of DNA transposons (4.3 *vs.* 0.6%). The region of overlap between two repeat fragments is classified as “Overlapping” if the two repeats belong to different repeat classes.

To ascertain the extent to which repetitive elements contribute to the expansion of the *D. ananassae* F element, we examined the analysis regions using three *de novo* repeat finders [*Red* (Girgis 2015), *WindowMasker* (Morgulis *et al.* 2006), and *Tallymer* (Kurtz *et al.* 2008)] that can identify novel repeat sequences. This analysis shows that repetitive sequences are major contributors to the expansion of the *D. ananassae* F element; the *D. ananassae* F element [*D. ana*: F (all)] has a repeat density of 56.4–74.5% compared to 14.4–29.5% for the *D. melanogaster* F element (Figure 2A). Within each species, the F element shows higher repeat density than the euchromatic region at the base of the D element.

Subsequent analyses using *tantan* (Frith 2011), *TRF* (Benson 1999), and *RepeatMasker* with *Drosophila* transposons in the RepBase library (Jurka *et al.* 2005; Smit *et al.* 2013) show that transposons are major contributors to the expansion of the *D. ananassae* F element. The *tantan* analysis results show that the *D. melanogaster* and *D. ananassae* analysis regions have a similar density of simple repeats (6.0–7.5%; Figure 2B). However, the *TRF* analysis shows that the *D. ananassae* F element has a higher density of tandem repeats than the *D. melanogaster* F element (5.6 *vs.* 2.8%; Figure 2C). *RepeatMasker* analysis using the *Drosophila* RepBase library shows that the *D. ananassae* F element has a transposon density of 78.6%, compared to 28.0% on the *D. melanogaster* F element (Figure 2D). Most of the increase in transposon density can be attributed to LTR (42.1 *vs.* 5.5%) and LINE retrotransposons (21.8 *vs.* 3.8%). By contrast, while the total sizes of the Helitron and DNA transposon remnants on the *D. ananassae* F element are greater than those on the *D. melanogaster* F element, the Helitron and DNA transposon density are lower on the *D. ananassae* F element than the *D. melanogaster* F element (7.3 *vs.* 10.4% and 5.6 *vs.* 7.6%, respectively).

To mitigate the impact of potential biases in the RepBase repeat library on the results of the transposon density estimates, we performed an additional repeat analysis using a more comprehensive repeat library. This centroid repeat library is comprised of the *Drosophila* RepBase library; the consensus sequences for *Drosophila* helentrons and Helentron-associated INterspersed Elements (HINE) (Thomas *et al.* 2014); and consensus sequences derived from structural, assembly, alignment, and k-mer based *de novo* repeat finders (see “Supplemental Methods” in File S7 for details). *RepeatMasker* analysis of the *D. ananassae* F element using this centroid repeat library results in a transposon density estimate of 90.0%, an increase of 11.4% compared to the *Drosophila* RepBase library. However, the distributions of the different transposon classes are similar to the results obtained using the *Drosophila* RepBase library (Figure S3 in File S7) (*RepeatMasker* results for the centroid repeat library and for each *de novo* repeat library are available on the GEP UCSC Genome Browser).

Consistent with the results of the repeat density analysis, eight of the 10 most common transposons on the *D. melanogaster* F element are classified as rolling circle (RC) Helitrons or DNA transposons (Table 2). The 589 copies of *DNAREP1* (*DINE-1*) RC/Helitron (from *D. simulans*, *D. melanogaster*, and *D. yakuba*) account for 32.1% of the transposon remnants on the *D. melanogaster* F element that were identified using *RepeatMasker*. The 84 copies of the *1360* DNA transposon (*PROTOP*) and its derivatives (*i.e.*, *PROTOP_A* and *PROTOP_B*) account for 12.0% of all the transposon remnants on the *D. melanogaster* F element. The *1360* sequence has previously been demonstrated to be a target for heterochromatin formation and associated silencing (Sun *et al.* 2004; Haynes *et al.* 2006).

**Table 2:** The 10 most common transposons (by the cumulative size of the transposon fragments) on the *D. melanogaster* F element [*i.e.*, the region between the most proximal (*JYalpha*) and the most distal (*Cadps*) genes]

Repeat	Total Size (bp)	Repeat Count	Repeat Class	Total Region (%)	Total Repeat (%)
*DNAREP1_DSim*	49,801	258	RC/Helitron	4.0	14.2
*DNAREP1_DM*	46,551	241	RC/Helitron	3.7	13.3
*PROTOP_A*	21,635	33	DNA transposon	1.7	6.2
*DNAREP1_DYak*	16,266	90	RC/Helitron	1.3	4.6
*DMCR1A*	12,993	40	LINE	1.0	3.7
*PROTOP*	11,769	18	DNA transposon	0.9	3.4
*FB4_DM*	10,290	36	DNA transposon	0.8	2.9
*TC1_DM*	8942	26	DNA transposon	0.7	2.6
*PROTOP_B*	8678	33	DNA transposon	0.7	2.5
*Gypsy-1_DSim-I*	8207	28	LTR	0.7	2.3

Eight of the 10 most common transposons on the *D. melanogaster* F element are RC/Helitrons and DNA transposons. The repeat count for each transposon corresponds to the total number of transposon fragments reported by the *RepeatMasker* annotation file.

By contrast, nine of the 10 most common transposons on the *D. ananassae* F element are LINE and LTR retrotransposons (Table 3). The *CR1-2_DAn* LINE retrotransposon is the most prominent type of transposon, with 2292 copies accounting for 9.6% of all transposon remnants on the *D. ananassae* F element. The second most common transposon is the *Helitron-N1_DAn*, with 2927 copies accounting for 6.4% of all transposon remnants and 68.9% of all RC/Helitrons identified on the *D. ananassae* F element. Seven of the most common transposons are LTR retrotransposons in the *BEL* and *Gypsy* families, accounting for 16.6% of the transposon remnants on the *D. ananassae* F element. Collectively, the 10 most common transposons account for 55.7% of all transposon remnants on the *D. melanogaster* F element and 35.6% of all transposon remnants on the *D. ananassae* F element.

**Table 3:** The 10 most common transposons (by the cumulative size of the transposon fragments) on all *D. ananassae* F-element scaffolds

Repeat	Total Size (bp)	Repeat Count	Repeat Class	Total Region (%)	Total Repeat (%)
*CR1-2_DAn*	1,326,170	2292	LINE	7.5	9.6
*Helitron-N1_DAn*	882,145	2927	RC/Helitron	5.0	6.4
*BEL-14_DAn-I*	599,914	754	LTR	3.4	4.3
*Loa-1_DAn*	424,975	546	LINE	2.4	3.1
*BEL-1_DBp-I*	396,161	868	LTR	2.2	2.9
*BEL-10_DAn-I*	291,535	573	LTR	1.7	2.1
*BEL-18_DAn-LTR*	264,131	551	LTR	1.5	1.9
*Gypsy-30_DAn-I*	256,665	277	LTR	1.5	1.9
*Gypsy-23_DAn-I*	243,223	362	LTR	1.4	1.8
*Gypsy-23_DAn-LTR*	240,787	443	LTR	1.4	1.7

Nine of the 10 most common transposons on the *D. ananassae* F element are LINE and LTR retrotransposons. The repeat count for each transposon corresponds to the total number of transposon fragments reported by the *RepeatMasker* annotation file.

In addition to analyzing the overall transposon distributions, we also compared the transposon distributions within the introns of coding spans and within the intergenic regions (Table S3 in File S7). The intronic regions of *D. melanogaster* F-element genes have a higher total transposon density than the intergenic regions (38.9 *vs.* 31.7%). The increase in transposon density within the intronic regions of *D. melanogaster* F-element genes can primarily be attributed to RC/Helitrons (+6.3%). By contrast, the intronic and intergenic regions of *D. ananassae* F-element genes have similar transposon density (78.1 *vs.* 80.0%). The intronic regions of *D. ananassae* F-element genes show lower density of LTR retrotransposons (−6.4%) compared to the intergenic region but a higher density of RC/Helitrons (+2.3%) and DNA transposons (+1.6%).

In both *D. ananassae* and *D. melanogaster*, the intronic regions of D-element genes show lower transposon density than the intergenic regions (6.9 *vs.* 20.4% and 5.3 *vs.* 9.9%, respectively). For *D. melanogaster*, most of the differences can be attributed to the lower density of LTR retrotransposons (−4.7%) within the intronic regions of D-element genes. However, the density of LINE retrotransposons is greater within the intronic regions (+2.1%) of *D. melanogaster* D-element genes than within the intergenic regions. For *D. ananassae*, all the major classes of transposons show lower density within the intronic regions than the intergenic regions, particularly for DNA transposons (−5.2%), LTR retrotransposons (−3.3%), and RC/Helitrons (−2.6%).

### Integration of Wolbachia sequences into the D. ananassae genome is a minor contributor to the expansion of the F element

Previous studies have shown that genomic sequences from *wAna* are integrated into the *D. ananassae* genome (Klasson *et al.* 2014; Choi *et al.* 2015). To ascertain the extent to which *wAna* integration has contributed to the expansion of the *D. ananassae* F element, we compared the *D. ananassae* improved assembly against the published assembly for *wAna*. Because the *wAna* assembly was based on Sanger sequencing reads recovered from the WGS sequencing of *D. ananassae* (Salzberg *et al.* 2005), the *wAna* assembly (GenBank assembly accession: GCA_000167475.1) is incomplete; it consists of 464 contigs with an N50 of 6730 bp (*i.e.*, contigs this size and larger account for half of the total length of the assembly). Hence, we also compared the *D. ananassae* genome against two additional *Wolbachia* species that have been manually improved to a single high-quality scaffold: *wRi* (Klasson *et al.* 2009b) and *wMel* (Wu *et al.* 2004).

**Figure 3:**
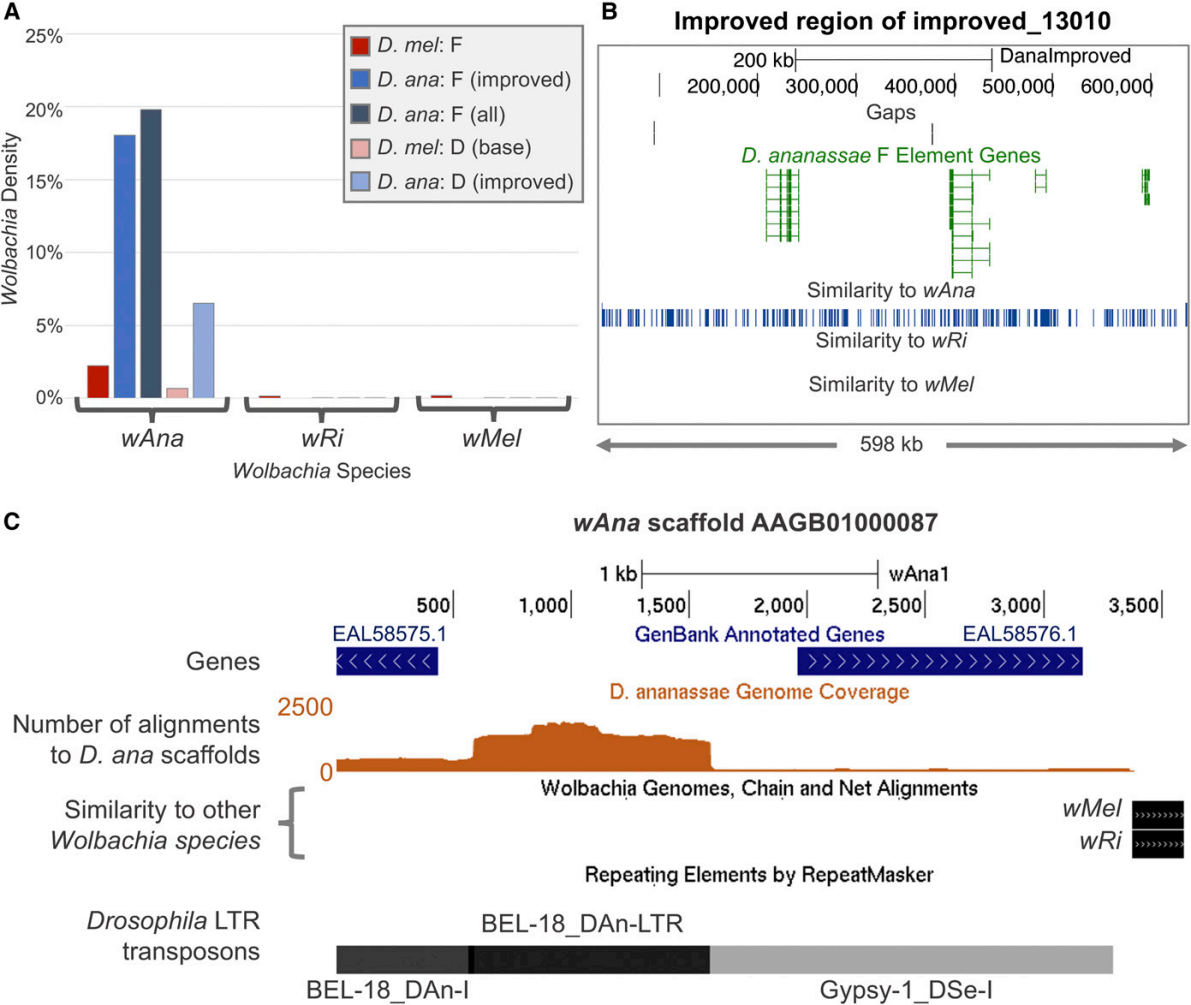
The high density of “*Wolbachia*” sequences in the *D. ananassae* F element can be attributed to *Drosophila* transposons in the *wAna* assembly. (A) *RepeatMasker* analysis shows that 19.8% of the *D. ananassae* F element matches the genome assembly for *wAna*. By contrast, 0.02% of the *D. ananassae* F element matches the genome assemblies for *wRi* and *wMel*. Similarly, the *D. ananassae* D element and the *D. melanogaster* F and D elements show a substantially higher density of regions that exhibit sequence similarity to the *wAna* assembly (0.68–6.51%) than the *wRi* and *wMel* assemblies (0.00–0.18%). (B) Distribution of regions with matches to the *wAna*, *wRi*, and *wMel* assemblies in the manually improved region of the *D. ananassae* F-element scaffold improved_13010. The matches to the *wAna* assembly are distributed throughout the improved scaffold (blue boxes) but there are no matches to either the *wRi* or the *wMel* assemblies. (C) The portions of the 3.6-kb *wAna* scaffold AAGB01000087 (*x*-axis) with large numbers of alignments to *D. ananassae* scaffolds show similarity to *Drosophila* transposons. The portions of the *wAna* scaffolds that show similarity to *D. ananassae* scaffolds were extracted from the *RepeatMasker* output and collated into an alignment coverage track relative to the *wAna* assembly (brown graph). Whole-genome Chain and Net alignments show that only the last 216 bp of this *wAna* scaffold has sequence similarity to the *wRi* and *wMel* assemblies. *RepeatMasker* analysis using the *Drosophila* RepBase library shows that the first 3.4 kb of this *wAna* scaffold has sequence similarity to the internal and long terminal repeat portions of the *BEL-18* LTR retrotransposon (*BEL-18_DAn-I* and *BEL-18_DAn-LTR*), as well the internal portion of the *Gypsy-1* LTR retrotransposon from *D. sechellia* (*Gypsy-1_DSe-I*). Most of the alignments can be attributed to *BEL-18_DAn-LTR*, with a maximum of 1835 alignments between the *wAna* scaffold AAGB01000087 and the *D. ananassae* scaffolds.

*RepeatMasker* comparison of the *wAna* genome assembly against the *D. ananassae* analysis regions suggest that *Wolbachia* sequences account for 19.79% of the *D. ananassae* F element and 6.51% of the base of the *D. ananassae* D element (Figure 3A). However, while earlier works have demonstrated that *wRi* is closely related to *wAna* (Klasson *et al.* 2009b; Choi and Aquadro 2014), *RepeatMasker* detected substantially fewer matches to *wRi* than to *wAna* on the *D. ananassae* F element (0.02%) and on the D element (0.05%). Similarly, we find substantially more matches to *wAna* than *wMel* on the *D. melanogaster* F and D elements (2.23 *vs.* 0.18% and 0.68 *vs.* 0.04%, respectively). Some of the matches to *wRi* and *wMel* can be attributed to *Wolbachia* genes that contain the same conserved domains (*e.g.*, *NADH dehydrogenase I*, *D subunit*) as *Drosophila* genes (*e.g.*, *ND-49*; Figure S4 in File S7).

To ensure that the discrepancies in the *Wolbachia* density estimates cannot be explained by misassemblies in the *D. ananassae* assembly, we examined the distribution of the regions that showed sequence similarity to the *wAna*, *wRi*, and *wMel* assemblies on the manually improved region of the *D. ananassae* F-element scaffold improved_13034. We find that regions that match the *wAna* assembly are distributed throughout the improved scaffold, but there are no matches to either the *wRi* or the *wMel* assemblies (Figure 3B). Collectively, these results indicate that the *wAna* assembly contains sequences that are classified as being part of the *Wolbachia* genome that have no sequence similarity to either *wRi* or *wMel*.

Comparison of the *wAna* matches with the *Drosophila* transposon matches identified using *RepeatMasker* shows that most of the *wAna* matches overlap with *Drosophila* transposons in the RepBase library. For *D. ananassae*, 85.0% of the *wAna* matches on the F element and 83.1% of the *wAna* matches on the D element overlap with *Drosophila* transposon remnants identified using *RepeatMasker*. The RC/Helitron *Helitron-N1_DAn* is the most prominent class of transposon that overlaps with the *wAna* matches, accounting for 25.0% of all *wAna* matches on the *D. ananassae* F element and 32.1% of all *wAna* matches on the D element. Similarly, 92.3% of the *wAna* matches on the *D. melanogaster* F element and 93.6% of the *wAna* matches on the D element overlap with *Drosophila* transposons remnants identified using *RepeatMasker*. The *DNAREP1_DSim* RC/Helitron most frequently overlaps with the *wAna* matches on the *D. melanogaster* F element (32.2%), while the *BEL_I-int* LTR retrotransposon most frequently overlaps with the *wAna* matches on the D element (19.9%).

Because the *wAna* assembly is constructed from *D. ananassae* WGS sequencing reads and the *wAna* genome is integrated into the *D. ananassae* genome (Klasson *et al.* 2014; Choi *et al.* 2015), *Drosophila* transposons could have been incorporated into the *wAna* assembly. Matches to these *Drosophila* transposons in the *wAna* assembly would inflate the apparent *wAna* density on the *D. ananassae* F and D elements compared to the densities of *wRi* and *wMel*. To test this hypothesis, we determined the portions of the *wAna* assembly that aligned to the *D. ananassae* assembly from the *RepeatMasker* output and then calculated the *D. ananassae* scaffold alignment coverage relative to the *wAna* assembly. We also searched the *wAna* assembly against the *Drosophila* RepBase library to identify *Drosophila* transposons within the *wAna* assembly.

The scaffold AAGB01000209 in the *wAna* assembly shows the highest *D. ananassae* alignment coverage among all the *wAna* scaffolds (maximum coverage = 4813). *RepeatMasker* analysis of this scaffold with the *Drosophila* RepBase library shows that 56% (818/1460 bp) of this scaffold exhibits sequence similarity to the RC/Helitron *Helitron-N1_DAn*. By contrast, this scaffold did not show any sequence similarity to either *wRi* or *wMel*. The presence of the *Helitron-N1_DAn* within scaffold AAGB01000209 would explain the large number of overlapping matches between the *wAna* scaffolds and *Helitron-N1_DAn* on the *D. ananassae* F and D elements.

Another *wAna* scaffold that shows high *D. ananassae* alignment coverage (max coverage = 1835) is AAGB01000087 (Figure 3C). *RepeatMasker* analysis using the *Drosophila* RepBase library shows that 91.5% (3292/3598 bp) of this scaffold has sequence similarity to the *BEL* and *Gypsy* LTR retrotransposons in *Drosophila*. Only the last 216 bp of this scaffold show sequence similarity to *wRi* and *wMel*; there is no *D. ananassae* alignment coverage in this region.

Collectively, our analyses suggest that the high density of *wAna* matches on the *D. ananassae* F and D elements can likely be attributed to *Drosophila* transposons in the *wAna* assembly. Using the manually improved *wRi* assembly as a reference, only 0.02% (3567 bp) of the *D. ananassae* F element and 0.05% (817 bp) of the base of the D element show similarity to *wRi*. Hence our results suggest that the integration of *Wolbachia* into the *D. ananassae* genome is not a major contributor to the expansion of the assembled portions of the *D. ananassae* F element.

### D. ananassae F element genes show distinct characteristics

To examine the impact of the expansion of the F element on gene characteristics, we used violin plots (Hintze and Nelson 1998) and the KW test (Kruskal and Wallis 1952) to compare the characteristics of the coding regions of F- and D-element genes in *D. ananassae* and *D. melanogaster*. If the KW test rejects the null hypothesis that the gene characteristics in the four analysis regions are derived from the same distribution, then *post hoc* DT (Dunn 1964) are used to identify the pairs of analysis regions that show significant differences [type I error (α) = 0.05]. The Holm–Bonferroni method is used to correct for multiple testing in the DT (Holm 1979) (see File S3 for the p-values of all the KW tests and the adjP for the DT).

**Figure 4:**
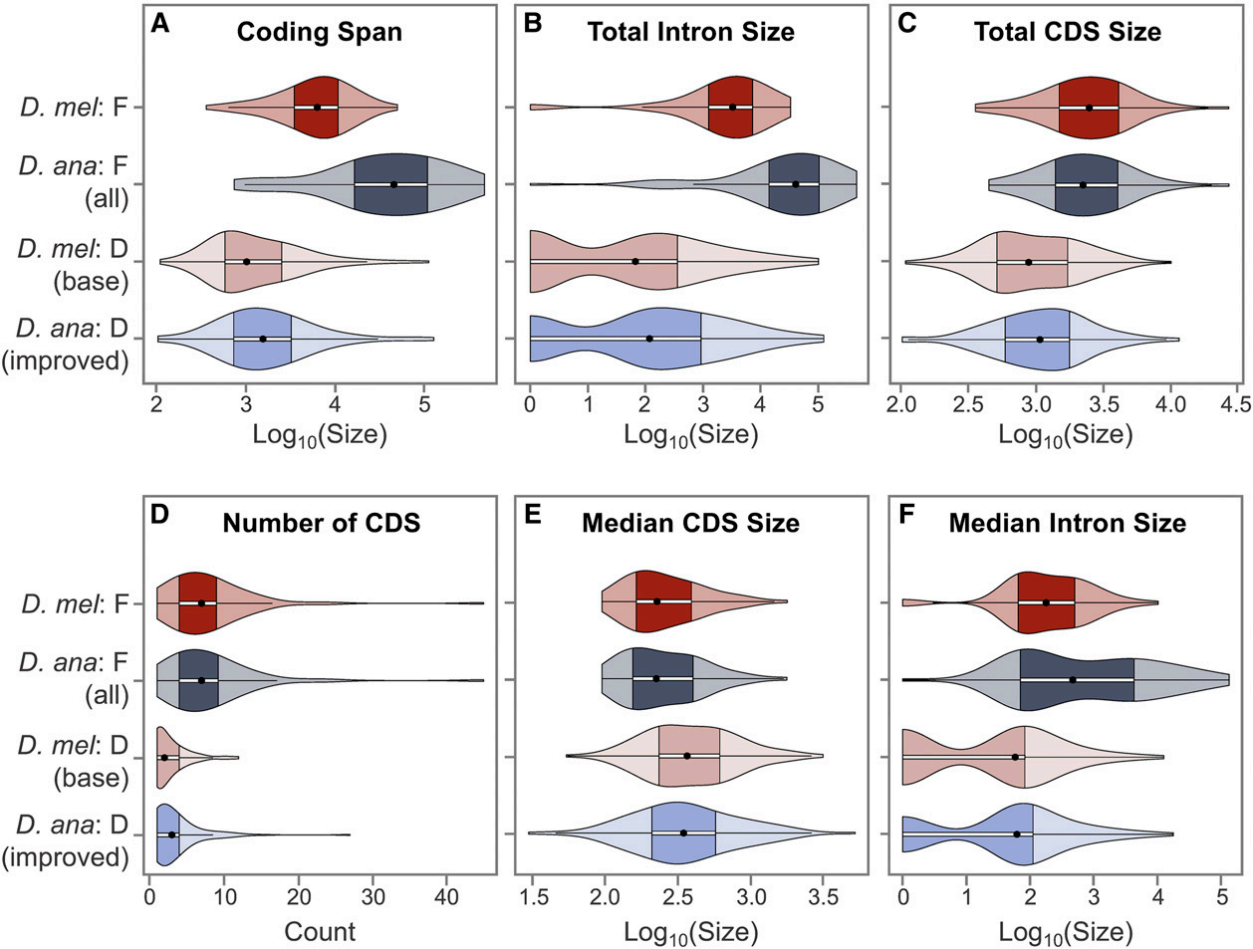
F-element genes show distinct gene characteristics compared to D-element genes. Each violin plot is comprised of a box plot and a kernel density plot. The ● in each violin plot denotes the median and the darker region demarcates the IQR, which spans from the first (Q1) to the third (Q3) quartiles. The whiskers extending from the darker region spans from Q1 = −1.5 × IQR to Q3 = +1.5 × IQR; data points beyond the whiskers are classified as outliers. (A) *D. ananassae* F-element genes have larger coding spans (start codon to stop codon, including introns) than *D. melanogaster* F-element genes. (B) The *D. ananassae* F-element genes have larger coding spans because they have larger total intron sizes than *D. melanogaster* F-element genes. (C) F-element genes have larger total coding exon (CDS) sizes than D-element genes in both *D. ananassae* and *D. melanogaster*. (D) F-element genes have more CDS than D-element genes. (E) F-element genes have smaller median CDS size than D-element genes. (F) The median intron size for *D. ananassae* and *D. melanogaster* F-element genes shows a bimodal distribution; this distribution pattern indicates that the expansion of the coding spans of *D. ananassae* F-element genes compared to *D. melanogaster* F-element genes can be attributed to the substantial expansion of a subset of introns.

The violin plot and KW test show that the coding span sizes (*i.e.*, distance from start codon to stop codon, including introns) of the genes in the four analysis regions have significantly different distributions (Figure 4A; KW test p-value = 1.40E−35). The DT show that the F-element genes in *D. ananassae* have significantly larger coding span sizes than in *D. melanogaster* (DT adjP = 6.82E−05). F-element genes in both *D. melanogaster* and *D. ananassae* have larger coding spans than D-element genes. Although the identity of the genes found at the base of the *D. ananassae* D element differs from those found at the base of the *D. melanogaster* D element (due to inversions and other rearrangements that have occurred within the element), DT shows that the difference in the distribution of coding span sizes is not statistically significant.

The difference in the distribution of coding span sizes can primarily be attributed to the differences in total intron sizes within the coding span (Figure 4B; p-value = 3.52E−37) and total coding exon (CDS) sizes (Figure 4C; p-value = 5.34E−17). DT shows that the coding spans of F-element genes in both *D. melanogaster* and *D. ananassae* are larger than D-element genes primarily because they have significantly larger total intron sizes (adjP range from 5.74E−30 to 2.88E−10) and total CDS sizes (adjP range from 3.69E−12 to 1.51E−07). Genes on the *D. ananassae* F element have significantly larger total intron sizes than *D. melanogaster* F element genes (adjP = 6.72E−05), while the differences in total CDS sizes are not statistically significant (adjP = 3.84E−01).

The larger total CDS sizes of F-element genes compared to D-element genes can be attributed to the greater number of CDS (Figure 4D; p-value = 5.08E−26; adjP range from 2.94E−17 to 1.26E−11). However, the median CDS sizes of F-element genes are significantly smaller than D-element genes (Figure 4E; p-value = 5.07E−06; adjP range from 1.07E−04 to 3.69E−03). In accordance with the larger total intron sizes in F-element genes, F-element genes have larger median intron sizes than D-element genes (Figure 4F; p-value = 5.68E−19; adjP range from 2.19E−11 to 1.39E−09). Although the violin plot shows that *D. ananassae* F-element genes have a larger IQR (first to third quartiles; IQR = 69–4286 bp) compared to *D. melanogaster* F-element genes (IQR = 64–499 bp), DT shows that this difference is not statistically significant (adjP = 4.11E−02). The kernel density component of the violin plots shows that the median intron sizes follow a bimodal distribution. Because the first quartile of the median intron sizes of *D. ananassae* F-element genes are similar to *D. melanogaster* F-element genes (69 *vs.* 64 bp) but the third quartile is 8.6 times larger (4286 *vs.* 499 bp), the expansion of the coding spans of *D. ananassae* F-element genes compared to *D. melanogaster* can primarily be attributed to the expansion of a subset of the introns.

In addition to comparing gene characteristics across the four analysis regions, we also compared the characteristics of *D. ananassae* F-element genes (minuend) with their orthologs in *D. melanogaster* (subtrahend) (D-element genes were omitted from this analysis because different sets of genes are found near the base of the *D. ananassae* and *D. melanogaster* D elements). These difference violin plots show that *D. ananassae* F-element genes generally have larger coding spans (median difference = +32,500 bp) than their *D. melanogaster* orthologs (Figure S5A in File S7) because they have larger total intron sizes (median difference = +32,570 bp; Figure S5B in File S7). By contrast, the total coding exon size is slightly smaller in *D. ananassae* F-element genes compared to their *D. melanogaster* orthologs (median difference = −15 bp; Figure S5C in File S7). In most cases, *D. ananassae* and *D. melanogaster* F-element genes have the same number of CDS (Q1 = median = Q3 = 0; Figure S5D in File S7). *D. ananassae* genes have similar median CDS sizes (median difference = 0 bp; Figure S5E in File S7) and larger median intron sizes (median difference = +213 bp; Figure S5F in File S7) compared to their *D. melanogaster* orthologs. These results indicate that the expansion of *D. ananassae* F-element genes compared to their *D. melanogaster* orthologs can be attributed to the increase in intron size.

### D. ananassae F-element genes show less optimal codon usage

As previously demonstrated, F-element genes in different *Drosophila* species show lower codon-usage bias than genes in other autosomes (Vicario *et al.* 2007; Leung *et al.* 2015). To assess how the expansion of the *D. ananassae* F element affects the usage of synonymous codons, we analyzed the distributions of codon usage bias for F- and D-element genes using two metrics: the Nc (Wright 1990) and the CAI (Sharp and Li 1987). Nc measures the deviations from the uniform usage of synonymous codons, and these deviations can be attributed to either mutational biases or selection. Nc values range from 20 to 61, where greater Nc values correspond to lower codon bias because it indicates that more synonymous codons were used. By contrast, CAI measures deviations from optimal codon usage, which reflects selection. CAI values range from 0.0 to 1.0, where greater values correspond to more optimal codon usage.

**Figure 5:**
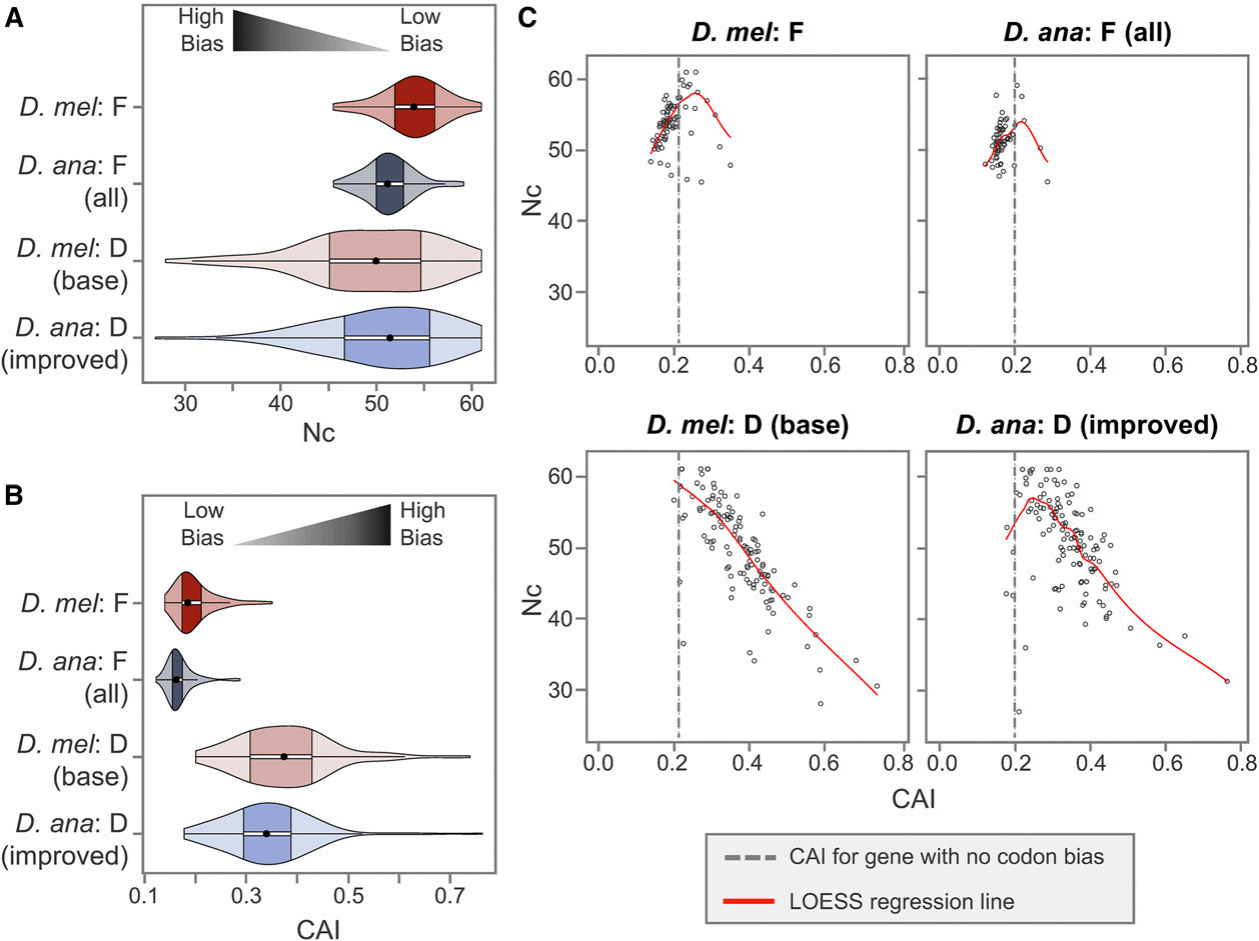
Codon bias in *D. ananassae* F-element genes can primarily be attributed to mutational biases instead of selection. (A) Violin plots of the Nc show that *D. ananassae* F-element genes exhibit stronger deviations from equal usage of synonymous codons (lower Nc) than *D. melanogaster* F-element genes. (B) Violin plots of the CAI show that *D. ananassae* F-element genes exhibit less optimal codon usage (lower CAI) than *D. melanogaster* F-element genes. F-element genes in both *D. ananassae* and *D. melanogaster* show less optimal codon usage (lower CAI) than D-element genes. (C) Scatterplot of Nc *vs.* CAI suggests that the codon bias in most *D. ananassae* and *D. melanogaster* F-element genes can be attributed to mutational bias instead of selection, as indicated by the placement of most of the genes in the portion of the LOESS regression line (red line) with a positive slope. By contrast, the codon bias in most *D. ananassae* and *D. melanogaster* D-element genes can be attributed to selection, as denoted by the LOESS regression line with a negative slope. The dotted line in each Nc *vs.* CAI scatterplot corresponds to the CAI value for a gene with equal codon usage relative to the species-specific reference gene sets constructed by the program *scnRCA* (0.200 for *D. ananassae* and 0.213 for *D. melanogaster*). Hence this species-specific threshold corresponds to the CAI value when the strengths of mutational bias and selection on codon bias are the same. A smaller percentage of F-element genes in *D. ananassae* (6/64; 9.4%) have CAI values above this species-specific CAI threshold compared to *D. melanogaster* (18/79; 22.8%).

Violin plots of Nc show that *D. ananassae* F-element genes exhibit significantly more deviations from the uniform usage of synonymous codons (*i.e.*, a lower Nc) than *D. melanogaster* F-element genes (Figure 5A; KW test p-value = 2.59E−06; DT adjP = 4.65E−04). The difference violin plot for Nc shows that most *D. ananassae* F-element genes have a lower Nc than their corresponding *D. melanogaster* orthologs (Figure S5G in File S7; median = −3.012). While the IQR for the Nc distribution of *D. ananassae* F-element genes (49.99–52.85) is smaller than the IQR for the *D. melanogaster* and the *D. ananassae* D-element genes (45.11–54.66 and 46.67–55.58, respectively), this difference is not statistically significant (adjP = 3.37E−01 and 3.83E−01, respectively).

Violin plots of CAI show that *D. ananassae* F-element genes exhibit significantly less optimal codon usage (*i.e.*, lower CAI) than *D. melanogaster* F-element genes (Figure 5B; p-value = 7.32E−55; adjP = 1.24E−02). The difference violin plot for CAI shows that this difference can be seen on a gene-by-gene basis; most *D. ananassae* F-element genes have a lower CAI than their corresponding *D. melanogaster* orthologs (Figure S5H in File S7; median = −0.025). Genes on the F element show significantly lower CAI compared to D-element genes in both *D. melanogaster* and *D. ananassae* (adjP range from 8.41E−38 to 1.96E−20).

To further investigate the factors that contribute to the differences in codon bias between *D. ananassae* and *D. melanogaster* F-element genes, we constructed Nc *vs.* CAI scatterplots for the four analysis regions (Vicario *et al.* 2007) and then applied LOESS (Cleveland and Devlin 1988) to capture the trends in each scatterplot (Figure 5C). Deviations from the uniform usage of synonymous codons (*i.e.*, lower Nc) can be attributed either to selection or mutational biases. By contrast, optimal codon usage (*i.e.*, higher CAI) can primarily be attributed to selection. Hence, the codon bias for genes that are placed in the part of the LOESS regression line with a positive slope can primarily be attributed to mutational biases, while the codon bias for genes that are placed in the part of the LOESS regression line with a negative slope can be attributed to selection (see Vicario *et al.* 2007 for details on this analysis technique).

The LOESS regression lines for the Nc *vs.* CAI scatterplots for the *D. melanogaster* and *D. ananassae* F elements both show an inverted-V pattern (Figure 5C, top). Most of the F-element genes are placed in the part of the LOESS regression line with a positive slope, which suggests that the codon bias in these genes can primarily be attributed to mutational biases. By contrast, most of the genes on the *D. ananassae* and *D. melanogaster* D elements are placed in the part of the LOESS regression line with a negative slope, which suggests that most of the codon bias for these genes can be attributed to selection (Figure 5C, bottom). Consistent with the results of the violin plot, *D. ananassae* F-element genes (Figure 5C, top right) show lower Nc values than *D. melanogaster* F-element genes (Figure 5C, top left). This pattern suggests that the additional codon bias in *D. ananassae* F-element genes can primarily be attributed to mutational biases instead of selection.

Earlier works have shown that mutational bias favors A/T while selection favors G/C at synonymous sites in *Drosophila* (Moriyama and Powell 1997; Vicario *et al.* 2007). Examination of the GC content of codons shows that F-element genes have a lower GC content than D-element genes (38.9–41.9% *vs.* 54.7–55.3%, Table 4). The most prominent difference in GC content is at the third position of the codon, where the GC content is 30.1% lower in *D. melanogaster* F-element genes and 34.4% lower in *D. ananassae* F-element genes, compared to the D-element genes in each species. The codons for *D. ananassae* F-element genes have the lowest GC content among all the analysis regions. In particular, the GC content of the third position of the codon is 5.8% lower in *D. ananassae* F-element genes compared to *D. melanogaster* F-element genes. By contrast, the GC content of the third codon position is only 1.5% lower in *D. ananassae* D-element genes compared to *D. melanogaster* D-element genes. Similarly, the GC content of the fourfold degenerate sites is 5.0% lower in *D. ananassae* F-element genes compared to *D. melanogaster* F-element genes, but only 2.0% lower in D-element genes. This pattern of lower G/C content of the wobble base in *D. ananassae* F-element genes compared to *D. melanogaster* F-element genes is seen in all amino acids that are encoded by two or more codons. The biggest difference in codon usage between *D. ananassae* and *D. melanogaster* F-element genes is histidine; with an 8.6% increase in the usage of CAT (69.6 *vs.* 61.0%) instead of CAC (30.4 *vs.* 39.0%) in *D. ananassae* (see “Supplemental Methods” in File S7 and File S4 for the comparisons of the codon frequencies for all codons).

**Table 4:** Codon GC content for *D. melanogaster* and *D. ananassae* F- and D-element genes

Metric	*D. mel*: F (%)	*D. ana*: F (All) (%)	*D. mel*: D (%)	*D. ana*: D (Improved) (%)
Coding GC	41.9	38.9	55.3	54.7
First letter GC	48.8	46.9	56.8	55.9
Second letter GC	40.2	39.0	42.7	42.9
Third letter GC	36.5	30.7	66.6	65.1
4D sites GC*^a^*	33.2	28.2	62.5	60.5

F-element genes show lower GC content than D-element genes in both *D. melanogaster* and *D. ananassae*. *D. ananassae* F-element genes show the lowest overall GC content among the genes in the four analysis regions, particularly at the third position of the codon.

a The “4D sites GC” corresponds to the GC content at fourfold degenerate sites (*i.e.*, the GC content at the third position of the codons that code for alanine, glycine, proline, threonine, and valine).

To further explore the relationships between CAI and other gene characteristics, we analyzed the gene characteristics of the subset of F-element genes that show high CAI values. Among the 64 *D. ananassae* F-element genes analyzed in this study, six genes (*Arf102F*, *ATPsynbeta*, *CG31997*, *Crk*, *ND-49*, and *RpS3A*) have higher CAI values than that of a gene with equal codon usage (*i.e.*, CAI > 0.200). Only five *D. ananassae* F-element genes (*Arf102F*, *ATPsynbeta*, *Crk*, *onecut*, and *RpS3A*) exhibit smaller total intron size compared to their *D. melanogaster* orthologs. Four of these genes (*Arf102F*, *ATPsynbeta*, *Crk*, and *RpS3A*) are present in both groups (Figure S6 in File S7, top right). Hence, the subset of *D. ananassae* F-element genes with a smaller total intron size compared to their *D. melanogaster* orthologs is significantly enriched in genes with higher CAI values (hypergeometric distribution p-value = 1.15E−04).

By contrast, of the 18 *D. melanogaster* F-element genes with CAI values greater than the CAI for a gene with equal codon usage (CAI > 0.213), only three genes (*ATPsynbeta*, *onecut*, and *RpS3A*) show a smaller total intron size in *D. ananassae* than in *D. melanogaster* (Figure S6 in File S7, top left; hypergeometric distribution p-value = 7.49E−02). The 16 *D. ananassae* F-element genes that show the largest increase in total intron size (*i.e.*, genes in the fourth quartile; size difference ≥ +97,610 bp) all have lower CAI values than a gene with uniform codon usage in *D. melanogaster* (Figure S6 in File S7, bottom left) and in *D. ananassae* (Figure S6 in File S7, bottom right). These results suggest that there is a small group of *D. ananassae* F-element genes that are under selection for more optimal codon usage and smaller coding span size.

### F element genes have lower T_m_ than D-element genes

**Figure 6:**
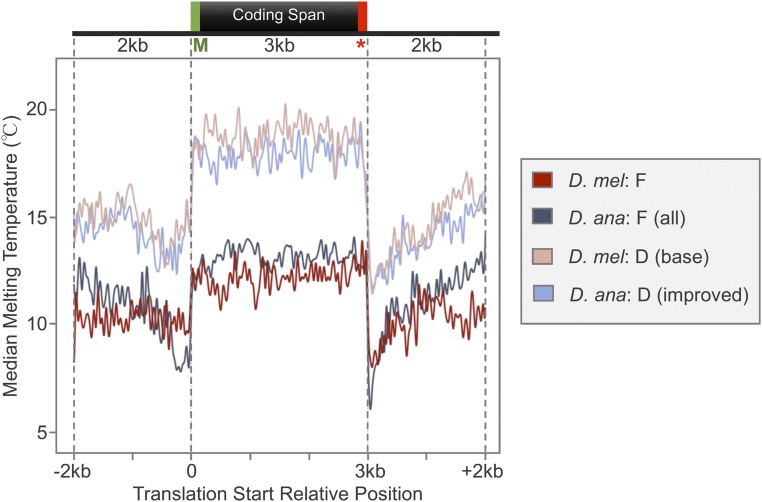
Metagene analysis shows that the coding spans of F-element genes have lower median 9-bp T_m_ than the genes at the base of the D element. The T_m_ profiles were determined using a 9-bp sliding window with a step size of 1 bp. The metagene consists of the 2-kb region upstream and downstream of the coding span, with the length of the coding spans normalized to 3 kb. While the codons in *D. ananassae* F-element genes have lower GC content, *D. ananassae* F-element genes exhibit a T_m_ profile that is similar to the *D. melanogaster* F-element genes. The green “M” below the coding span denotes the Methionine at the translation start site, and the red star denotes the stop codon.

Given the prior observations of low T_m_ across the coding spans of F-element genes (Leung *et al.* 2015), we constructed median T_m_ metagene profiles for the coding spans of the genes in all four analysis regions (Figure 6). The metagene profiles show that F-element genes have lower T_m_ than D-element genes in *D. ananassae* (median = 13.15 *vs.* 18.00°) and in *D. melanogaster* (median = 12.27 *vs.* 18.75°). Despite the lower codon GC content and the higher transposon density within the introns of *D. ananassae* F-element genes (both of which have been inferred to lower T_m_), the coding spans of *D. ananassae* F-element genes show slightly higher median T_m_ than the median T_m_ of *D. melanogaster* F-element genes (median = 13.15 *vs.* 12.27°; IQR = 12.54–13.74° *vs.* 11.50–13.00°). Hence these two characteristics are not the primary determinant of the T_m_ of the coding span. The metagene profiles also show that the 2-kb regions upstream and downstream of the coding spans have lower T_m_ than the coding span in all four analysis regions.

### F-element genes maintain distinct histone modification profiles

To explore the epigenomic landscape of the *D. ananassae* F element, we generated ChIP-Seq data sets for three histone modifications using third instar larvae: dimethylation of lysine 4 on histone 3 (H3K4me2), associated with transcription start sites during gene activation; dimethylation of lysine 9 on histone 3 (H3K9me2), associated with heterochromatin formation and gene silencing; and trimethylation of lysine 27 on histone 3 (H3K27me3), associated with gene silencing through the Polycomb system. The modENCODE project has previously produced ChIP-Seq data for these histone modifications in *D. melanogaster* (Ho *et al.* 2014), thereby enabling us to compare the epigenomic landscape of the *D. ananassae* and *D. melanogaster* F elements.

**Figure 7:**
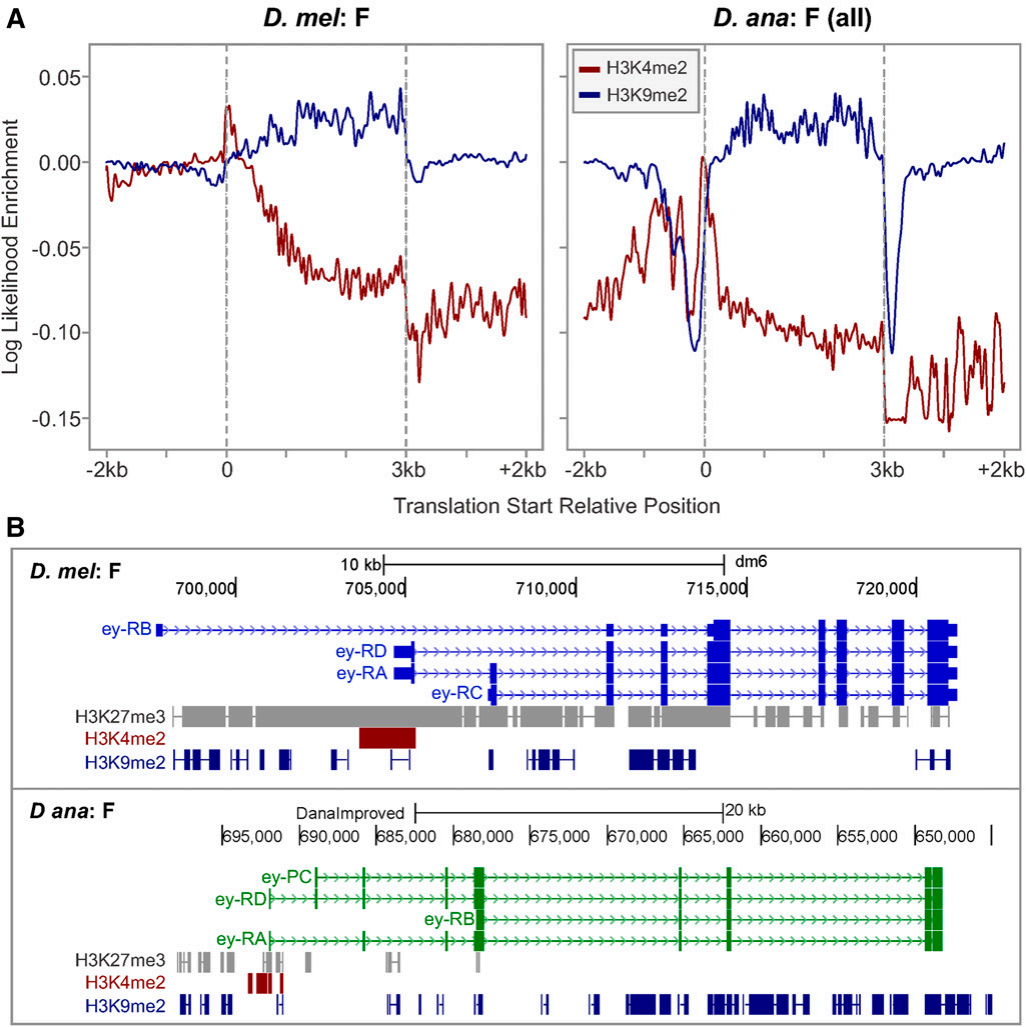
Histone modification profiles for *D. ananassae* and *D. melanogaster* F-element genes at the third instar larval stage of development. (A) Metagene analysis shows that the region surrounding the 5′ end of F-element genes is enriched in H3K4me2 while the body of the coding span is enriched in H3K9me2. The values in the *y*-axis within each metagene plot correspond to the log-likelihood ratio between each ChIP sample and input control (assuming a dynamic Poisson model) as determined by *MACS2*. (B) Differences in the H3K27me3 enrichment patterns for the *D. melanogaster ey* gene and its ortholog in *D. ananassae*. The entire coding span of the *ey* gene is enriched in H3K27me3 in *D. melanogaster* (top) (for the *D. melanogaster* gene models, the thick boxes denote the coding exons and the thin boxes denote the untranslated regions). By contrast, only the region surrounding the 5′ end of the *ey* ortholog in *D. ananassae* shows H3K27me3 enrichment. The 5′ ends of the A and D isoforms of *ey* shows enrichment of H3K4me2 and H3K27me3 in both *D. melanogaster* and *D. ananassae*. These bivalent domains suggest that these two isoforms of *ey* are poised for activation at the third instar larval stage of development in both species.

Because the gene annotations for *D. ananassae* only include the coding regions, the analysis of histone-modification enrichment patterns are relative to the translated regions instead of the transcribed regions, even though the H3K4me2 modification typically shows enrichment near the transcription start sites of active genes (reviewed in Boros 2012). Metagene analysis of the H3K4me2 and H3K9me2 log-enrichment profiles produced by *MACS2* (Zhang *et al.* 2008) show that *D. ananassae* and *D. melanogaster* F-element genes exhibit similar H3K4me2 and H3K9me2 enrichment profiles (Figure 7A). The 5′ end of F-element genes are enriched in H3K4me2 while the coding span is enriched in H3K9me2. By contrast, consistent with a previous report on the epigenomic landscape of euchromatic genes in *D. melanogaster* (Riddle *et al.* 2011), D-element genes in both species show H3K4me2 enrichment near the 5′ end of the gene but the coding span generally does not show H3K9me2 enrichment.

A subset of *Drosophila* genes show enrichment of H3K27me3; the expression of these genes is typically regulated by Polycomb group (PcG) proteins (reviewed in Lanzuolo and Orlando 2012). Eight of the *D. melanogaster* F-element genes (*dati*, *ey*, *fd102C*, *fuss*, *Sox102F*, *sv*, *toy*, and *zfh2*) show H3K27me3 enrichment at the third instar larval stage, with this histone mark being broadly distributed throughout the coding span of each gene (Figure S7 in File S7, left). Four of these genes (*dati*, *fuss*, *sv*, and *zfh2*) show stronger H3K27me3 enrichment near the 5′ end of the gene. The corresponding orthologs of these eight genes on the *D. ananassae* F element also show H3K27me3 enrichment. However, in all cases, the H3K27me3 enrichment tends to be restricted to the region near the 5′ end of the gene while the body of the coding span is enriched in H3K9me2 (Figure S7 in File S7, right).

Six of the eight F-element genes (*ey*, *fd102C*, *Sox102F*, *sv*, *toy*, and *zfh2*) that show H3K27me3 enrichment also show H3K4me2 enrichment in the region surrounding the 5′ end of the gene in both *D. melanogaster* and *D. ananassae* (Figure S7 in File S7). These “bivalent promoters” (*i.e.*, promoters with both active and repressive histone modifications) are often found in developmentally regulated genes that are poised to be activated upon differentiation (reviewed in Voigt *et al.* 2013). For the *D. melanogaster* gene *ey* and its *D. ananassae* ortholog, H3K4me2 is only enriched in the region surrounding the 5′ ends of the A and D isoforms (Figure 7B), which suggests that these two isoforms of *ey* are poised to be activated at the third instar larval stage of development in both species.

### D. ananassae F-element and D-element genes exhibit similar expression profiles

To assess whether the unusual genomic landscape of the *D. ananassae* F element would affect the levels of gene expression, we analyzed the RNA-Seq data from adult females and adult males (Chen *et al.* 2014), female carcass, male carcass, female ovaries, male testes (Rogers *et al.* 2014), and embryos (Kumar *et al.* 2012). This expression analysis is based on release 1.04 of the *D. ananassae Gnomon* gene predictions from FlyBase (Attrill *et al.* 2016), which include predictions of untranslated regions and alternative isoforms.

**Figure 8:**
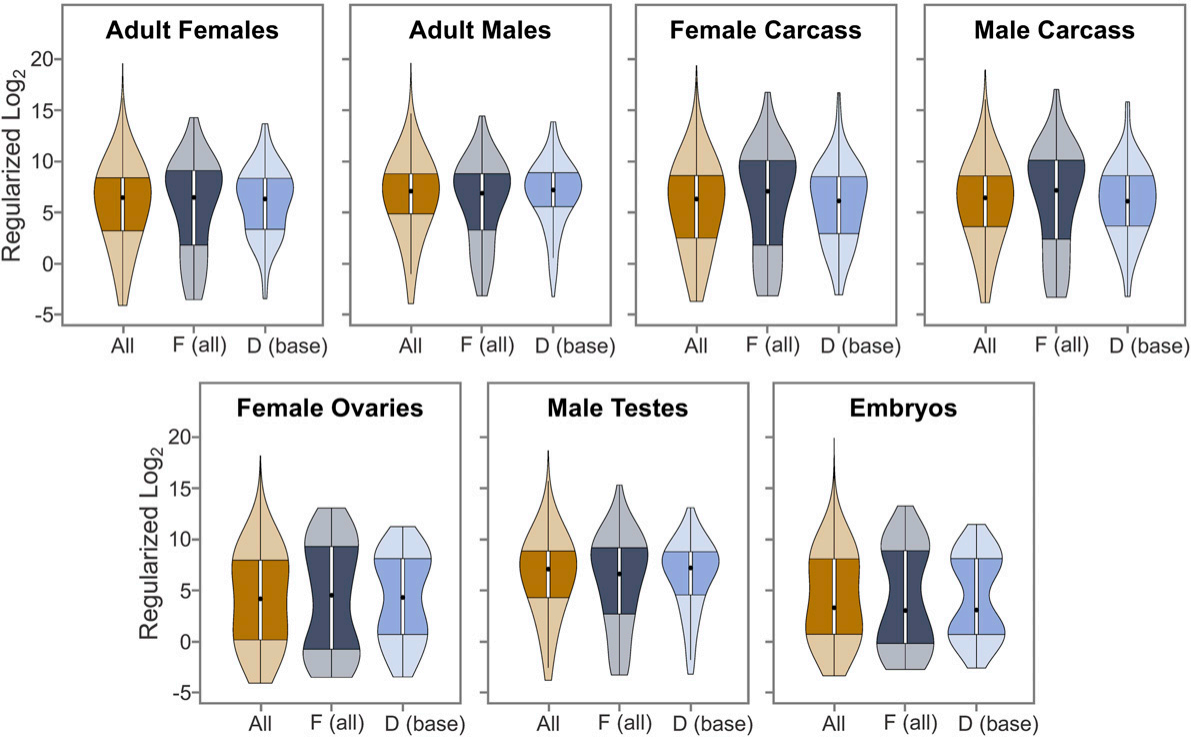
*D. ananassae* F-element genes show similar expression patterns compared to genes on other Muller elements. RNA-Seq reads from seven samples (adult females, adult males, female carcass, male carcass, female ovaries, male testes, and embryos) were mapped against the improved *D. ananassae* genome assembly and the read counts for the *Gnomon* gene predictions were tabulated by *htseq-count*. The read counts for the seven samples were normalized by library size and then transformed using Tikhonov/ridge regularization in the *DESeq2* package to stabilize the variances among the samples. The violin plots compare the distributions of the regularized log_2_ expression values for the *D. ananassae Gnomon* gene predictions on all scaffolds (All), on the F-element scaffolds [F (all)], and on the base of the D element [D (base)] for these different developmental stages and tissues.

Violin plots of the rlog-transformed expression values show that the *Gnomon*-predicted genes on the *D. ananassae* F element exhibit similar expression patterns compared to genes on all scaffolds and genes at the base of the D element (Figure 8). The KW test shows that the differences in expression patterns among these three regions are not statistically significant in each of the seven developmental stages and tissues (p-values range from 1.30E−01 in female carcass to 9.50E−01 in embryos). Thus, despite the unusual genomic and epigenomic landscape, *D. ananassae* F-element genes show a range of expression patterns that is similar to those of genes in euchromatic regions.

However, preliminary analyses of the seven RNA-Seq samples indicate a negative Spearman’s rank correlation coefficient between CAI and rlog expression values for *D. ananassae* F-element genes, but a positive correlation for all *D. ananassae* genes (“Supplemental Results,” Figure SM1, and Table SM1 in File S7). These results are consistent with the hypothesis that mutational biases (rather than selection) are the primary contributors to the codon biases observed in *D. ananassae* F-element genes. However, the results are considered preliminary because of the small number of replicates available for each RNA-Seq sample, and potential issues with the *Gnomon* gene predictions (*e.g.*, *GF22695*; Figure SM2 in File S7).

Our preliminary results also suggest that *D. ananassae* and *D. melanogaster* F-element genes exhibit similar ranges of expression values despite the substantial expansion of most *D. ananassae* F-element genes compared to their *D. melanogaster* orthologs (“Supplemental Results,” Figure SM3, and Table SM2 in File S7). Collectively, these preliminary results suggest that *D. ananassae* F-element genes have adapted to their local environment in some way to maintain their expression in a heterochromatic domain with high repeat density. See “Supplemental Results” and “Supplemental Methods” in File S7 for additional details on these analyses.

## Discussion

This study describes an initial survey of the characteristics of the *D. ananassae* F element through a comparative analysis with the *D. melanogaster* F element and the base of the *D. ananassae* and *D. melanogaster* D elements. In concordance with the results of previous comparative analysis of *D. virilis* (Leung *et al.* 2010) and of *D. erecta*, *D. mojavensis*, and *D. grimshawi* (Leung *et al.* 2015), we find that the *D. ananassae* F element generally exhibits distinct characteristics compared to the base of the D element. However, the contrasts between the characteristics of the *D. ananassae* F and D elements tend to be larger than the contrasts seen in the other *Drosophila* species, presumably because of the higher density of repetitious elements in the *D. ananassae* F element.

Given the propensity for repetitive sequences to be misassembled in whole-genome assemblies (Salzberg and Yorke 2005; Phillippy *et al.* 2008), we first assessed the veracity of the F-element scaffolds in the *D. ananassae* CAF1 assembly prior to the analysis of the repeat and gene characteristics. Manual sequence improvement of 1.4 Mb of the *D. ananassae* F element and 1.7 Mb of the base of the D element resolved 35 gaps in the CAF1 assembly (Table 1) and resolved a major misassembly within the *D. ananassae* F-element scaffold improved_13034 (Figure 1). In conjunction with the manually curated gene models, we have generated data that provide an important metric for assessing the quality of the F-element scaffolds in the *D. ananassae* CAF1 assembly and provide more accurate measures of repeat and gene characteristics. Because the comparisons of the improved regions and the corresponding regions in the CAF1 assembly show that they are generally in congruence with each other (Figure 1A), where possible we expanded the general analysis to include all putative F-element scaffolds in the *D. ananassae* CAF1 assembly to produce a more comprehensive overview of the properties of the *D. ananassae* F element.

Repeat analysis of the F element and the base of the D element from *D. ananassae* and *D. melanogaster* show that the expansion of the *D. ananassae* F element can primarily be attributed to repetitive sequences (Figure 2A). The *D. ananassae* F element shows a similar density of simple repeats compared to the other analysis regions (Figure 2B), and a higher density of tandem repeats (Figure 2C). However, most of the increase in total repeat density can be attributed to transposons, particularly LTR and LINE retrotransposons (Figure 2D).

The transposon density estimate for the *D. ananassae* F element (78.6%) is substantially higher than the previous estimate of 32.5% (Schaeffer *et al.* 2008). However, older versions of the *Drosophila* RepBase library has been shown to have a strong bias toward transposons in *D. melanogaster*, which would result in an underestimate of the transposon density in the other *Drosophila* species (*Drosophila* 12 Genomes Consortium 2007; Leung *et al.* 2010). Hence, the difference in the transposon-density estimates can be attributed to the use of a newer version of the *Drosophila* RepBase library (release 20150807), which includes transposons from other *Drosophila* species, including *D. ananassae*. Repeat analysis of the *D. ananassae* F element using the centroid repeat library (comprised of *Drosophila* repeats in the RepBase library, helentrons and HINE sequences, and repeats from *de novo* repeat finders) results in a repeat density estimate of 90.0% (Figure S3 in File S7), which suggests that there might be additional *D. ananassae* transposons that are not part of the RepBase library.

The repeat analysis also shows substantial differences in the repeat composition of the *D. melanogaster* and *D. ananassae* F elements. In contrast to the *D. melanogaster* F element where eight out of 10 of the most common transposons are RC/Helitrons and DNA transposons (Table 2), nine out of 10 of the most common transposons on the *D. ananassae* F element are LTR and LINE retrotransposons (Table 3). The two most prominent transposons on the *D. ananassae* F element are the LINE element *CR1-2_DAn* and the RC/Helitron *Helitron-N1_DAn*, which account for 7.5 and 5.0% of the *D. ananassae* F-element scaffolds, respectively.

An unusual aspect of the expansion of the *D. ananassae* F element is that the expansion appears to be mostly limited to a single Muller element, as *D. melanogaster* and *D. ananassae* have similar total genome sizes (Gregory and Johnston 2008). Previous analyses have shown that the accumulation of retrotransposons can result in a substantial increase in genome size (Kidwell 2002), particularly in plant genomes (Piegu *et al.* 2006; reviewed in Lee and Kim 2014). However, a recent invasion of retrotransposons that results in the expansion of the *D. ananassae* F element should also result in a concomitant increase in the size of the euchromatic portion of the genome. While the base of the *D. ananassae* D element shows a 6.7% increase in transposon density compared to the *D. melanogaster* D element (+6.7%; 14.4 *vs.* 7.7%), this increase in transposon density is much smaller than the increase of 50.6% seen on the F element (78.6 *vs.* 28.0%). Because the amplifications of transposons could impact the fitness of the organism (reviewed in Pritham 2009), one possible explanation for this dichotomy is that the F element already has the necessary mechanisms in place for silencing transposons and for allowing gene function in a region with a high density of transposons (Sun *et al.* 2004; Riddle *et al.* 2012). These mechanisms would mitigate the impact of a local expansion of transposons on the fitness of *D. ananassae* and enable the F element to accumulate a higher density of transposons than the euchromatic regions.

Two earlier studies have shown that the *wAna* genome is integrated into the genome of multiple strains of *D. ananassae* via horizontal gene transfer (Klasson *et al.* 2014; Choi *et al.* 2015). However, our analyses indicate that the integration of *wAna* into the *D. ananassae* genome is unlikely to be a major contributor to the expansion of the assembled portions of the *D. ananassae* F element (Figure 3). *RepeatMasker* analysis shows that the *D. ananassae* F element has a high density of regions that show sequence similarity to the *wAna* assembly (Figure 3A). However, while previous studies have shown that *wAna* is closely related to *wRi* (Klasson *et al.* 2009b; Choi and Aquadro 2014), we find that most of the regions within the *D. ananassae* F element that show strong sequence similarity to the published *wAna* assembly do not show sequence similarity to either the *wRi* or the *wMel* assemblies (Figure 3B).

The large disparity in the density of matches to the *wAna* assembly compared to the *wRi* and *wMel* assemblies on the *D. ananassae* F element could be attributed to the different strategies used to construct these *Wolbachia* assemblies. The subset of genomic reads from the WGS sequencing of *D. ananassae* that shows sequence similarity to *wMel* and their corresponding mate-pair reads was used to construct the *wAna* assembly (Salzberg *et al.* 2005). By contrast, the *wRi* (Klasson *et al.* 2009b) and the *wMel* (Wu *et al.* 2004) assemblies have been manually improved to high quality. A previous study has identified *Drosophila* retrotransposons within *wAna* fragments that are integrated into the *D. ananassae* genome (Choi *et al.* 2015). Hence, constructing the *wAna* assembly based on the *D. ananassae* WGS reads would likely result in the incorporation of *Drosophila* transposons into the *wAna* assembly. Consistent with this hypothesis, we find that most of the matches between the *wAna* assembly and the *D. ananassae* assembly show sequence similarity to transposons in the *Drosophila* RepBase library (Figure 3C). However, given the potential for *Wolbachia* to act as a vector for the horizontal transfer of transposable elements (Dupeyron *et al.* 2014), it is still possible that these *Drosophila* transposons are actually part of the *wAna* genome.

Using sequence similarity to *wRi* and *wMel* as the metric, we found only a low density of *Wolbachia* sequences within the 1.4-Mb manually improved region of the *D. ananassae* F element and the collection of all 18.7-Mb F-element scaffolds from the *D. ananassae* assembly (Figure 3A). However, because of gaps and misassemblies in the improved assembly, the possibility remains that *wAna* is integrated into the unassembled portions of the *D. ananassae* F element. In contrast to *wAna*, both *wRi* and *wMel* appear to infect but are not integrated into the *D. simulans* and *D. melanogaster* genomes. Hence the *wAna* genome might contain additional sequences that facilitate horizontal gene transfer that are not in either *wRi* or *wMel*. However, an improved *wAna* assembly would be required to identify these novel sequences. Based on the data currently available, we conclude that the expansion of the assembled portions of the *D. ananassae* F element can be explained by the increase in the density of *Drosophila* transposons, and that the integration of *wAna* is only a minor contributor to the expansion of the *D. ananassae* F element.

Previous work has demonstrated that F-element genes exhibit distinct characteristics compared to genes in the euchromatic reference regions in *D. melanogaster*, *D. erecta*, *D. virilis*, D. *mojavensis*, and *D. grimshawi* (Leung *et al.* 2010, 2015). Part of the difference in gene characteristics can be attributed to the low rates of recombination on the F element, which reduces the effectiveness of selection (Hill and Robertson 1966; Betancourt *et al.* 2009; Arguello *et al.* 2010). To assess the impact of the expansion of the *D. ananassae* F element on gene characteristics, we compared the characteristics of the genes on the *D. melanogaster* and *D. ananassae* F elements and on a euchromatic reference region at the base of the D elements. The base of the D element is used as a comparison region to mitigate the confounding effects caused by the proximity to the centromere, where there is a lower rate of homologous recombination and most of the homologous recombination can be attributed to gene conversions instead of crossing over (Shi *et al.* 2010; reviewed in Talbert and Henikoff 2010).

Our analyses show that *D. ananassae* F-element genes generally maintain the same contrast to D-element genes as seen in *D. melanogaster* (Figure 4), but for some features the differences are more pronounced. F-element genes have larger coding spans, primarily because they have larger total intron size, and this is particularly true for the *D. ananassae* F-element genes. This result is consistent with previous analysis that shows large introns tend to appear in regions with low rates of recombination (Carvalho and Clark 1999). The bimodal median intron size distribution suggests that the larger coding spans of *D. ananassae* F-element genes can be attributed to the expansion of a subset of introns. Most of the increase in total intron size for *D. ananassae* F-element genes compared to *D. melanogaster* F-element genes can be attributed to the higher density of transposable elements within introns.

Previous analysis has shown that *D. melanogaster* genes located in regions that exhibit lower rates of recombination have longer coding regions and more coding exons (Comeron and Kreitman 2000). Hence, our results are consistent with the hypothesis that, similar to the *D. melanogaster* F element, the *D. ananassae* F element has a low rate of recombination. We also find that, despite having smaller median CDS sizes, genes on both the *D. melanogaster* and *D. ananassae* F element have larger total CDS size because they have more coding exons than D element genes.

Low rates of recombination have also been associated with more uniform usage of synonymous codons (*i.e.*, lower codon bias) in *D. melanogaster* (Kliman and Hey 1993). Codon bias can primarily be attributed to mutational bias and selection (reviewed in Hershberg and Petrov 2008; Plotkin and Kudla 2011). Earlier studies have suggested that highly expressed genes tend to show the strongest codon bias because selection tends to favor codons that pair with the most abundant tRNAs (Moriyama and Powell 1997; reviewed in Angov 2011). More recent studies suggest that, in addition to affecting gene expression, codon bias could also affect secondary structures in mRNA, the efficacy of protein translation, and protein folding (reviewed in Novoa and Ribas de Pouplana 2012).

Using the analysis technique developed by Vicario *et al.* (2007), we compared the codon usage patterns of F- and D-element genes in *D. melanogaster* and *D. ananassae* using two metrics: the Nc and the CAI. Our results show that F-element genes exhibit more uniform usage of synonymous codons (higher Nc) (Figure 5A) and less optimal codon usage (lower CAI) than D-element genes (Figure 5B). The codon bias for most F-element genes can primarily be attributed to mutational bias instead of selection (Figure 5C). *D. ananassae* F-element genes show stronger deviations from uniform usage of synonymous codons than *D. melanogaster* F-element genes, but this bias can primarily be attributed to mutational biases instead of selection.

Previous studies have demonstrated that mutation in *Drosophila* has a bias toward A/T, whereas selection tends to favor G/C at synonymous sites (Powell and Moriyama 1997; Vicario *et al.* 2007). Consistent with these observations, we find that the F-element genes have a lower GC codon content than D-element genes, particularly at the wobble base (Table 4). In concordance with the results of the Nc and CAI analysis, we find that codons in *D. ananassae* F-element genes have a lower GC content than *D. melanogaster* F-element genes. The lower GC content supports the hypothesis that the lower Nc exhibited by *D. ananassae* F-element genes can primarily be attributed to mutational bias.

Analysis of the five genes (*Arf102F*, *ATPsynbeta*, *Crk*, *onecut*, and *RpS3A*) on the *D. ananassae* F element that have smaller total intron sizes than their *D. melanogaster* orthologs shows that they tend to have a higher CAI than the CAI for a gene with uniform usage of synonymous codons (Figure S6 in File S7). Hence while most of the *D. ananassae* F-element genes are under weak selective pressure, a small subset of F-element genes is under stronger selective pressure to maintain their coding span size and to maintain a more optimal pattern of codon usage. These results are consistent with previous analysis in *D. melanogaster*, which shows a negative correlation between gene length and codon bias (Moriyama and Powell 1998).

Previous studies have shown that the stability of the 9-bp RNA–DNA hybrid within the elongation complex affects the efficacy of transcript elongation (Palangat and Landick 2001). Our previous analysis of the T_m_ metagene profiles in *D. melanogaster*, *D. erecta*, *D. mojavensis*, and *D. grimshawi* has shown that F-element genes exhibit lower T_m_ than D-element genes (Leung *et al.* 2015). Given that GC content is correlated with T_m_ (Frank-Kamenetskii 1971), and that *D. ananassae* F-element genes exhibit low codon GC content, we compared the T_m_ metagene profiles of F- and D-element genes in *D. ananassae* with the corresponding T_m_ metagene profiles from *D. melanogaster*. The analysis shows that *D. ananassae* and *D. melanogaster* F-element genes have similar T_m_ metagene profiles, such that F-element genes have similarly lower T_m_ compared to D-element genes in both species (Figure 6). These results indicate that neither the shift in codon GC content nor the increased transposon density within and around the F-element genes in *D. ananassae* are sufficient to shift the T_m_ metagene profiles, which suggests that the T_m_ must reflect other features of sequence organization. The lower T_m_ could facilitate the transcription of F-element genes and is consistently observed in the five *Drosophila* species studied to date.

Another unusual characteristic of the *D. melanogaster* F element is its distinct epigenomic landscape compared to the other autosomes (Kharchenko *et al.* 2011; Riddle *et al.* 2012). ChIP-Seq analysis of H3K4me2 and H3K9me2 from third instar larvae shows that most *D. ananassae* F-element genes exhibit histone-modification enrichment patterns similar to those seen in *D. melanogaster* F-element genes, where the region surrounding the 5′ end of the gene is enriched in H3K4me2 while the body of the coding span is enriched in H3K9me2 (Figure 7A). This histone modification pattern is consistent with the pattern seen in *D. melanogaster* heterochromatic genes (Yasuhara and Wakimoto 2008), and could enable the transcription machinery to access the core promoters of *D. ananassae* F-element genes while maintaining silencing of the transposons that reside within introns.

Many key genes involved in *Drosophila* development are activated by the Trithorax group of proteins and silenced by PcG proteins (reviewed in Grimaud *et al.* 2006; Schuettengruber *et al.* 2007). Regions enriched in H3K27me3 are recognized by the chromodomain of Polycomb (Min *et al.* 2003), resulting in gene silencing (reviewed in Blackledge *et al.* 2015). ChIP-Seq analyses of H3K27me3 show that the eight *D. melanogaster* F-element genes that exhibit H3K27me3 enrichment at the third instar larval stage also show H3K27me3 enrichment in the *D. ananassae* orthologs (Figure S7 in File S7). The H3K27me3 enrichment tends to be limited to the region surrounding the 5′ end of *D. ananassae* F-element genes, while the body of the coding span are enriched in H3K9me2 (Figure 7B).

Of the eight F-element genes that show H3K27me3 enrichment, six genes (*ey*, *fd102C*, *Sox102F*, *sv*, *toy*, and *zfh2*) also show H3K4me2 enrichment near the 5′ ends of the genes in both *D. melanogaster* and *D. ananassae*. Past studies of mammalian embryonic stem (ES) cells have identified promoters that show enrichments of both H3K27me3 and H3K4me3 (*i.e.*, bivalent domains). These genes are poised for activation upon the differentiation of the ES cells (Young *et al.* 2011; Nair *et al.* 2012; reviewed in Voigt *et al.* 2013). Hence, the histone-modification enrichment patterns for these six F-element genes suggest that they are poised for activation in both *D. melanogaster* and *D. ananassae* at the third instar larval stage of development.

Despite residing in a domain with high repeat density, *D. melanogaster* F-element genes show a similar fraction of active genes in S2 cells (∼50%) compared to genes in euchromatin (Riddle *et al.* 2012). Analysis of *D. ananassae* gene expression levels using RNA-Seq data from seven developmental stages and tissues shows that *D. ananassae* F-element genes exhibit a similar range of expression levels compared to all *D. ananassae* genes and compared to genes at the base of the D element (Figure 8). Thus, these genes are able to function while in the midst of a heterochromatic domain with high repeat density.

Past studies have shown that heterochromatic genes in *D. melanogaster*, such as *lt* (Wakimoto and Hearn 1990) and *rl* (Eberl *et al.* 1993), depend on the properties of the heterochromatic environment for proper expression. Inversely, euchromatic genes that are relocated to a heterochromatic region exhibit partial gene silencing (*i.e.*, position effect variegation; reviewed in Elgin and Reuter 2013). Comparative analysis of genes (*e.g.*, *lt* and *Yeti*) that have moved between heterochromatic and euchromatic domains in different *Drosophila* species show that the structure of the core promoter for these genes is typically conserved (Yasuhara *et al.* 2005; Moschetti *et al.* 2014). These observations suggest that the expression of heterochromatic genes might depend on specialized enhancers within heterochromatic domains (reviewed in Yasuhara and Wakimoto 2006; Corradini *et al.* 2007).

This study demonstrates that transposons can be an important contributor to the expansion of a chromosome, and provides insights into the impact on the resident genes. Our study shows that *D. ananassae* F-element genes can continue to be expressed at similar levels despite the large increase in repetitive content. Understanding the mechanisms that enable *D. ananassae* F-element genes to be expressed within a highly repetitive heterochromatic domain could provide insights into the factors that regulate gene expression in other eukaryotic genomes with high repeat density, such as in plant and mammalian genomes.

## Supplementary Material

Supplemental material is available online at www.g3journal.org/lookup/suppl/doi:10.1534/g3.117.040907/-/DC1.
