# Supplementary material for: Retrotransposons Are the Major Contributors to the Expansion of the Drosophila ananassae Muller F Element
Source: G3 (Bethesda). 2017 Jun 30;7(8):2439–60. doi: 10.1534/g3.117.040907 (PMC5555453; doi:10.1534/g3.117.040907)
Supplement: Supplementary file 7 [file 2439FileS7.docx]

# Supplemental Figures

Figure S1. Overview of the manually improved regions of the *D. ananassae* F and D elements scaffolds. Three regions from two *D. ananassae* F element scaffolds (improved_13034 and improved_13010) were manually improved; 94% of these regions (1.4Mb) were improved to high quality. One region from the *D. ananassae* D element scaffold (improved_13337) was manually improved; 98% of this region (1.7 Mb) was improved to high quality. Regions that have been manually improved are demarcated by teal boxes in the “Improved Regions” track. Within these regions, unresolved sub-regions are denoted by red boxes (Unresolved), sub-regions with *Phred* quality scores below 30 (i.e., estimated error rate greater than 1/1000 bases) are denoted by brown boxes (Low Quality), and sub-regions that were covered only by a single read are denoted by purple boxes (Single Subclone). The distribution of *Gnomon* gene predictions from FlyBase is shown in the “*Gnomon* Genes” track. The density of transposon remnants identified using *RepeatMasker* with the *Drosophila* RepBase repeat library is shown in the “Repeat Density” track (grayscale ranges from 0–100%, darker color denotes higher repeat density).

Figure S2. Using restriction digests and PacBio reads to verify the fosmid assemblies. (A) Comparison of the fragment sizes from the actual restriction digests with the *in-silico* digests for the fosmid project 1773K10. Top: the fragment sizes from the actual *Eco*RI digest for the fosmid 1773K10 (brown lines, left) were compared against the fragment sizes that were produced based on the locations of *Eco*RI restriction sites (GAATTC) in the final consensus sequence (*in-silico*; green lines, right). The fragment sizes for the *in-silico* digest should match those in the actual digest if the final assembly for the fosmid project is correct. Bottom: the sequence improvement criteria for *D. ananassae* requires at least two actual and *in-silico* digests to match. The digest window shows the comparison of the actual and the *in-silico* digests for *Hind*III (with the restriction site AAGCTT). The purple lines in the digest window denote multiple digest fragments with similar sizes (i.e., two fragments that are approximately 1.7kb each). (B) Using PacBio reads to confirm the assembly for the fosmid project 6173E04. Top: graphical overview of the *blastn* alignments of PacBio subreads against the improved consensus sequence for 6173E04. A subset of PacBio subreads that covered the same region as the fosmid were selected from the *BLASR* alignments of all PacBio subreads to the *D. ananassae* CAF1 assembly. The rectangles denote the alignment between the final fosmid sequence (query) to each PacBio subread (subject); the red color indicates these alignments have normalized bit scores that are greater than or equal to 200. Bottom: the dot plot alignment between each of the selected PacBio subreads (y-axis) and the improved consensus sequence for the fosmid (x-axis). The dot plots for each PacBio subread are shown in separate boxes and the black dots within each dot plot denote regions of sequence similarity between the PacBio subread and the improved consensus sequence. In some cases, the *SMRT analysis pipeline* failed to identify the SMRT bell adapter sequences within the polymerase reads. This error results in PacBio subreads that contain both the forward and the reverse complement sequences of the same genomic region (red arrow). Based on the distance and the orientation of the consistent forward-reverse mate pairs, we can distinguish these artifacts within the PacBio subreads from a genuine collapsed inverted repeat within the assembly. Overlapping PacBio subreads cover the entire fosmid project in a linear array, thereby confirming the assembly.

Figure S3. *RepeatMasker* repeat density estimates for all the analysis regions using a custom *Drosophila* transposon library. The transposon library consisted of transposon sequences in the *Drosophila* RepBase library, the helentrons and HINE elements consensus sequences, and the species-specific transposon sequences generated by five *de novo* repeat finders (*LTRHarvest* + *LTRdigest*, *RepeatModeler*, *ReAS*, *dnaPipeTE*, *Tedna*; see Supplemental Methods). The result of the transposon density analysis using this custom repeat library is generally consistent with the results obtained using the RepBase *Drosophila* library, whereby the *D. ananassae* F element shows much higher transposon density than the *D. melanogaster* F element (90.0% versus 28.2%). Most of the increase in the transposon density of the *D. ananassae* F element compared to the *D. melanogaster* F element can be attributed to the expansion of LTR (45.2% versus 5.6%) and LINE (20.9% versus 3.7%) retrotransposons.

Figure S4. Some of the matches between the *D. ananassae* genome and the *wMel* and *wRi* assemblies can be attributed to conserved protein domains within *Wolbachia* proteins. The *tblastn* alignments of the *Wolbachia NADH dehydrogenase I, D subunit* protein sequences against the *D. melanogaster* (left) and *D. ananassae* (right) F elements show significant sequence similarity to the *Drosophila ND-49* gene. These matches can be attributed to the conserved *Respiratory-chain NADH dehydrogenase, 49 Kd subunit* domain (Pfam accession number PF00346) within this *Wolbachia* gene (AAS14267.1 in *wMel*, ACN95184.1 in *wRi*, and EAL57867.1 in *wAna*). The additional regions of similarity between the *wAna* genome assembly and the *D. ananassae* F element overlap with transposon remnants that have been identified using *RepeatMasker* with the *Drosophila* RepBase library.

Figure S5. Violin plots of the differences in gene characteristics between *D. ananassae* F element (minuend) genes and their *D. melanogaster* orthologs (subtrahend). The darker region in each violin plot corresponds to the interquartile range and the black dot denotes the median. The grey dotted line corresponds to no difference between the *D. ananassae* gene and its *D. melanogaster* ortholog. The violin plots show that *D. ananassae* F element genes have larger coding spans (A) because they have larger total intron size (B). *D. ananassae* F element genes have smaller CDS sizes (C) than the corresponding *D. melanogaster* ortholog, but have similar number of coding exons (D) and median CDS size (E). Consistent with the larger total intron size, *D. ananassae* F element genes have larger median intron size compared to their *D. melanogaster* orthologs (F). *D. ananassae* F element genes have greater deviations from uniform usage of synonymous codons (lower Nc; G), but exhibit less optimal codon usage (lower CAI) compared to their *D. melanogaster* orthologs (H).

Figure S6. The *D. ananassae* F element genes that have smaller total intron size than their *D. melanogaster* orthologs tend to show more optimal codon usage. The Nc versus CAI scatterplot shows that the codon bias in most *D. melanogaster* (top left) and *D. ananassae* (top right) F element genes can be attributed to mutational biases instead of selection, as denoted by the LOESS regression line (red line) with a positive slope. The species-specific CAI value for a gene with equal codon usage relative to a reference gene set identified using *scnRCA* (0.200 for *D. ananassae* and 0.213 for *D. melanogaster*) is demarcated by the dotted line in each scatterplot. The blue dots denote the five *D. ananassae* F element genes that have a smaller total intron size than their *D. melanogaster* orthologs. Four out of these five genes have CAI values above the CAI value for a *D. ananassae* gene with equal usage of synonymous codons. These genes are placed in the part of the LOESS regression line with a negative slope, which indicates that the codon bias within these genes can primarily be attributed to selection. By contrast, the sixteen *D. ananassae* F element genes with the largest increase in total intron size compared to their orthologs in *D. melanogaster* (i.e., genes in the fourth quartile; orange dots) are all found within the part of the LOESS regression line with a positive slope) in both *D. melanogaster* (bottom left) and *D. ananassae* (bottom right). These results suggest that a small subset of *D. ananassae* F element genes are under selection that results in more optimal codon usage and smaller coding span size than the rest of the F element genes.

Figure S7. Histone modification enrichment profiles in 3^rd^ instar larvae for eight *D. melanogaster* F element genes that show H3K27me3 enrichment (left) compared with their *D. ananassae* orthologs (right). Six out of the eight *D. ananassae* and *D. melanogaster* genes (*ey*, *fd102C*, *Sox102F*, *sv*, *toy*, and *zfh2*) show enrichment of both H3K4me2 (dark red) and H3K27me3 (dark grey) in the region surrounding the 5’ end of the gene, which suggests that they are poised to be activated. The *dati* and *fuss* genes show only H3K27me3 and H3K9me2 (dark blue) enrichment, which suggests that these genes are inactive at this stage of development. (The *sv* ortholog in *D. ananassae* consists of two separate features because the region contains multiple gaps; as a consequence, some of the coding exons of *sv* are missing from the *D. ananassae* assembly.) The H3K27me3 domain tends to cover most of the coding span in *D. melanogaster*. By contrast, the H3K27me3 domains are restricted to the regions surrounding the 5’ end of the genes in the *D. ananassae* orthologs, while the body of the coding span is enriched in H3K9me2.

# Supplemental Tables

| Tool | Version |
| --- | --- |
| *ART* | 03-19-2015 |
| *BBMap* | 35.14 |
| *BEDTools* | 2.23.0 |
| *BLASR* | 1.3.1 (git commit 0087df7b) |
| *Bowtie* | 1.1.1 |
| *Bowtie 2* | 2.2.5 |
| *BWA* | 0.7.12-r1039 |
| *CD-HIT* | 4.6.4 |
| *CENSOR* | 4.2.29 |
| *Cufflinks* | 2.2.1 |
| *dnaPipeTE* | b0.31_20150224 |
| *EMBOSS* | 6.6.0 |
| *GenomeTools* | 1.5.5 |
| *Gepard* | 1.30 |
| *GNU Parallel* | 20150422 |
| *HISAT2* | 2.0.1-beta |
| *HMMER* | 3.1b2 |
| *HTSeq* | 0.6.1 |
| *LAST* | 556 |
| *MACS2* | 2.1.0.20150731 |
| *MUSIC* | git commit 8d5828a0 |
| *NCBI BLAST+* | 2.2.30+ |
| *Oases* | 0.2.09 |
| *PASTEC* | 1.0 |
| *Phred, Phrap, and Consed* | 0.071220.b, 1.090518, 25.0 |
| *Picard* | 1.130 |
| *R* | 3.2.3 |
| *R*: *Bioconductor* | 2.30.0 |
| *R*: *ChIPseeker* | 1.6.7 |
| *R*: *DEseq2* | 1.10.1 |
| *R*: *dunn.test* | 1.3.2 |
| *R*: *edgeR* | 3.12.0 |
| *R*: *fANCOVA* | 0.5-1 |
| *R*: *vioplot* | 0.2 |
| *RECON* | 1.08 |
| *Red* | 05/22/2015 |
| *RepeatMasker* | open-4.0.5 |
| *RepeatModeler* | open-1.0.8 |
| *RepeatScout* | 1.0.5 |
| *RepEnrich* | 1.2 |
| *REPET* | 2.2 |
| *SAMtools* | 1.2 |
| *scnRCA* | 04/11/2013 |
| *Sickle* | 1.33 |
| *SPALN* | 2.1.4 |
| *tantan* | 13 |
| *Tedna* | 1.2.2 |
| *TopHat* | 2.1.0 |
| *TRF* | 4.04 |
| *UCSC Genome Browser* | 324 |
| *USEARCH* | 8.0.1623 |
| *WU BLAST* | 2.0MP-WashU |

Table S1. Version information for the bioinformatic tools used in this study

| Label | Coordinates | First Gene | Last Gene |
| --- | --- | --- | --- |
| *D. mel*: F | chr4:930-1271129 | *JYalpha* | *Cadps* |
| *D. ana*: F (improved) | improved_13010:39105-636864 | *gw* | *ci* |
|  | improved_13034:476367-871682 | *CG2316* | *Ank* |
|  | improved_13034:1676206-2166988 | *Crk* | *lgs* |
| *D. mel*: D (base) | chr3L:21652271-22955285 | *CG43980* | *CG32461* |
| *D. ana*: D (improved) | improved_13337:20820934-22545932 | *Ets65A* | *SPoCk* |

Table S2. The genomic coordinates for the *D. melanogaster* and *D. ananassae* analysis regions. The *D. ananassae* F element analysis region is composed of one region from the scaffold improved_13010 and two regions from the scaffold improved_13034.

| Repeat Class | *D. mel*: F | *D. ana*: F (all) | *D. mel*: D (base) | *D. ana*: D (improved) |
| --- | --- | --- | --- | --- |
| % RC/Helitron | +6.3% | +2.3% | -1.0% | -2.6% |
| % DNA Transposons | +1.8% | +1.6% | -0.9% | -5.2% |
| % LINE | -1.4% | 0.0% | +2.1% | -1.3% |
| % LTR | +0.3% | -6.4% | -4.7% | -3.3% |
| % Overlapping | +0.3% | +0.5% | -0.1% | -0.6% |
| % Unclassified | -0.2% | +0.1% | 0.0% | -0.5% |
| Total | +7.1% | -1.9% | -4.6% | -13.5% |

Table S3. Differences in the density of each class of transposon between the intronic (minuend) and the intergenic (subtrahend) regions

# Supplemental Files

File S1. DanaImproved_noncanonical_features.xlsx

File S2. DanaImproved_putative_F_scaffolds.xlsx

File S3. DanaImproved_comprehensive_KWtest_stats.xlsx

File S4. DanaImproved_codon_usage_stats.xlsx

File S5. Participating_course_list.xlsx

File S6. Dananassae_paper_author_contributions.xlsx

# Supplemental Results

## Identifying *D. ananassae* F element scaffolds

Of the 79 *D. melanogaster* F element genes in FlyBase release 6.06, 76 can be placed in the *D. ananassae* assembly. The genes *Kif3C* and *RhoGAP102A* are missing from the assembly, while multiple copies of the gene *CG11231* are found in the *D. ananassae* assembly. [The matches to *CG11231* overlap with the *R1-2_DAn* LINE retrotransposon in the *Drosophila* RepBase library (Jurka *et al.* 2005).] The *D. ananassae* orthologs of two *D. melanogaster* F element genes (*CG11076* and *CG11077*) are found on scaffold_13117. Because most of the genes within this 5.79 Mb scaffold are orthologous to genes found on the *D. melanogaster* Muller A element, these two genes are likely located on the *D. ananassae* A element. We also found a gene (*CG4038*) from the *D. melanogaster* C element that is placed on the improved *D. ananassae* F element region improved_13034_2.

Of the remaining 74 *D. melanogaster* F element genes, we found six partial genes (*CG33978*, *JYalpha*, *eIF4G*, *pan*, *pho*, and *sv*) and five genes where the coding exons are distributed across multiple scaffolds (*Asator*, *CG1909*, *CG11148*, *Slip1*, and *dati*). These genes were excluded from further analysis. We also identified and corrected the consensus errors in three F element genes (*Dyrk3*, *Rfabg*, and *bip2*) that resulted in frame shifts and in-frame stop codons in the conceptual protein translations.

## Comparison of codon GC content

The amino acid that shows the biggest difference in codon usage between *D. ananassae* and *D. melanogaster* F element genes is histidine, which shows an 8.6% increase in the usage of CAT (69.6% versus 61.0%) instead of CAC (30.4% versus 39.0%) in *D. ananassae*. By contrast, D element genes in *D. ananassae* and *D. melanogaster* show similar usage of CAT (68.7% versus 70.1%) and CAC (31.3% versus 29.9%). Other amino acids that show a strong preference for A/T at the wobble base in *D. ananassae* F element genes compared to *D. melanogaster* F element genes include tyrosine (+8.4% for TAT over TAC), lysine (+8.0% for AAA over AAG), asparagine (+7.6% for AAT over AAC), and glutamine (+7.3% for CAA over CAG). The biggest difference in codon usage for *D. ananassae* and *D. melanogaster* D element genes is the stop codon, which shows a 6.3% increase in the usage of TAA (46.5% versus 40.2%) in *D. ananassae* D element genes compared to TAG (-4.0%; 27.1% versus 31.1%) and TGA (-2.3%; 26.4% versus 28.7%).

**Correlation between CAI and expression levels of *D. ananassae* F element genes**

Because selection tends to favor codons that pair with the most abundant tRNAs, past studies have shown a positive correlation between codon bias and gene expression levels [(Moriyama and Powell 1997), reviewed in Angov 2011]. However, in contrast to the genes on the base of the D element, the Effective Number of Codons (Nc) versus Codon Adaptation Index (CAI) scatterplot indicates that codon bias in most *D. ananassae* F element genes can be attributed to mutational biases instead of selection (Figure 5C). This difference might result in a weaker correlation between CAI and gene expression levels in F element genes.

To test this hypothesis, we calculated the correlation between CAI and the regularized log2 expression values (rlog) for seven RNA-Seq samples (Figure 8). We find that the CAI and rlog for *D. ananassae* F element genes show a negative Spearman’s rank correlation coefficient in the seven RNA-Seq samples. In contrast, the Spearman correlations between CAI and rlog for genes on the base of the D element and on all scaffolds in the *D. ananassae* genome assembly are generally positive (Table SM1). The only exception is the embryos sample for the base of the D element, which shows a slightly negative Spearman correlation (-0.001).

In all seven RNA-Seq samples, genes on the base of the D element show only a weak positive Spearman correlation between CAI and rlog. The adult females and adult males RNA-Seq samples show the highest Spearman correlations on the base of the D element (0.135 and 0.176, respectively), and on all scaffolds in the *D. ananassae* assembly (0.344 and 0.331, respectively). The Spearman correlations between CAI and rlog for genes on all scaffolds are greater than those for the base of the D element. This discrepancy might be attributed to the substantial differences in the number of genes in the two datasets (13,891 versus 117 genes).

| RNA-Seq Sample | *D. ana*: All  N = 13,891 | *D. ana*: F (all)  N = 114 | *D. ana*: D (base)  N = 117 |
| --- | --- | --- | --- |
| Adult Females | 0.344 | -0.348 | 0.135 |
| Adult Males | 0.331 | -0.334 | 0.176 |
| Female Ovaries | 0.212 | -0.329 | 0.003 |
| Male Testes | 0.169 | -0.336 | 0.020 |
| Female Carcass | 0.291 | -0.351 | 0.097 |
| Male Carcass | 0.288 | -0.352 | 0.066 |
| Embryos | 0.210 | -0.386 | -0.001 |

Table SM1 Spearman correlations between the Codon Adaptation Index (CAI) and the regularized log_2_ expression values (rlog) from seven *D. ananassae* RNA-Seq samples. The Spearman correlations were calculated for *D. ananassae Gnomon* gene predictions on all scaffolds in the *D. ananassae* assembly (All), on F element scaffolds [F(all)], and on the base of the D element [D (base)]. Spearman correlation instead of Pearson correlation was used to mitigate the impact of influential points (i.e., outliers).

Examination of the CAI versus rlog scatterplots confirmed the results of the Spearman correlation analyses (Figure SM1). The trends captured by the locally estimated scatterplot smoothing [LOESS (Cleveland and Devlin 1988)] in the CAI versus rlog scatterplots of F element genes show regression lines with a negative slope. By contrast, the LOESS lines show a positive slope for the *Gnomon* gene predictions on the base of the D element and on all scaffolds. Hence the available evidence indicates a negative correlation between CAI and rlog in *D. ananassae* F element genes. These results are consistent with the hypothesis that most of the codon bias in *D. ananassae* F element genes can be attributed to mutational biases instead of selection.


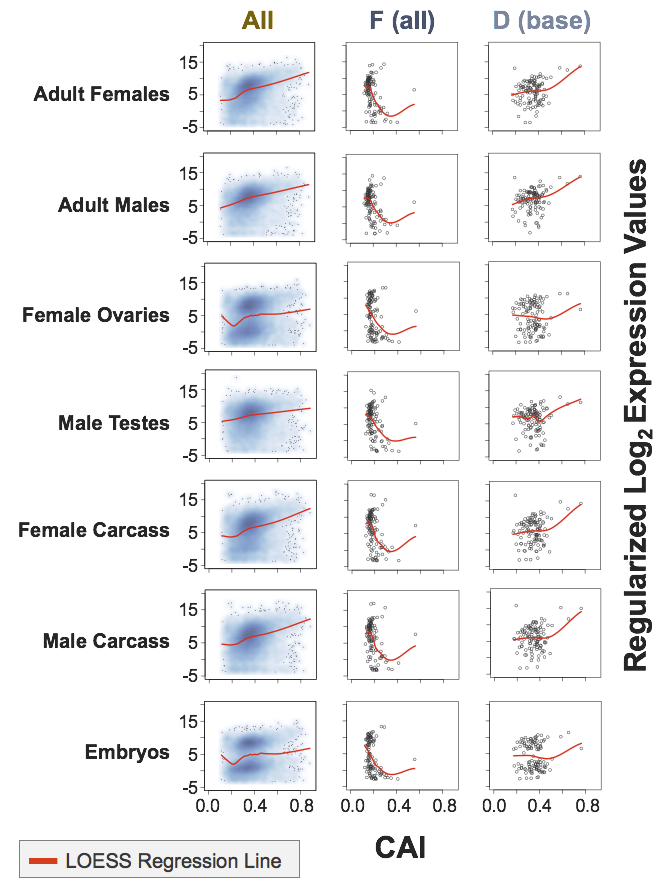


Figure SM1. The scatterplots of CAI (x-axis) versus rlog (y-axis) in seven RNA-Seq samples show a negative correlation between CAI and rlog in *D. ananassae* F element genes. The scatterplots show the CAI and rlog values for *Gnomon* gene predictions on all scaffolds (All), on all F element scaffolds [F (all)], and on the base of the D element [D (base)]. The red line within each scatterplot corresponds to the LOESS regression line. To avoid overplotting, smoothed color density scatterplots were used to show the CAI versus rlog values for the *Gnomon* gene predictions on all scaffolds, where the intensity of the blue color corresponds to the density of data points.

The scatterplots for the *Gnomon* gene predictions on the F element consistently show a single outlier (*GF22695*; FlyBase accession: FBgn0099689) that deviates from the negative correlation between CAI and rlog (Figure SM2A). This predicted gene is located on scaffold_13767, a 1.69 Mb scaffold that contains the putative orthologs of three F element genes (*CG1909*, *Sox102F*, and *CG11148*). The FlyBase gene report shows that *GF22695* contains a Isopropylmalate dehydrogenase-like domain (InterPro accession: IPR024084).

Figure SM2. *GF22695* on the *D. ananassae* F element scaffold_13767 corresponds to a partial gene duplication of the *CG5028* ortholog. (A) *GF22695* is an outlier in the CAI versus rlog scatterplot for the adult females RNA-Seq sample. It shows the highest CAI value (0.557) among all the *Gnomon* gene predictions on the *D. ananassae* F element scaffolds, and it has an intermediate expression level (rlog = 6.479) in the adult females RNA-Seq sample. (B) *GF22695* is located next to a 15.3 kb gap (SlateGray arrow). RNA-Seq read coverage from the adult females sample only covers part of the A isoform of *GF22695* (GF22695-RA). The purple arrows demarcate regions within GF22695-RA with low (< 10) RNA-Seq read coverage. The RepeatMasker track shows that GF22695-RA is flanked by two *Helitron-N1_DAn* helitrons. The *Helitron-N1_DAn* at scaffold_13767:1529548-1529807 was inserted into the middle of the *hAT-N1B_DBp* DNA transposon, indicating that the helitron insertion is a more recent event. (C) *CG5028* in *D. melanogaster* has 5 or 6 coding exons. A tblastn search of these coding exons against the *D. ananassae* scaffold_13767 shows that the second coding exon (CDS) of *GF22695* has a partial match to CDS 3_9229_0, and full length matches to CDS 4_9229_0, 5_9229_2, 6_9229_2, and 7_9229_1 of the *CG5028* gene in *D. melanogaster*.

Examination of the genomic region surrounding *GF22695* shows that this predicted gene is located next to a 15.3 kb gap, and it is flanked by two *Helitron-N1_DAn* helitrons (Figure SM2B). A blastp search of the GF22695-PA protein sequence against the FlyBase *D. melanogaster* “Annotated proteins” database shows that the best match to this feature is the *D. melanogaster* gene *CG5028* (FlyBase ID: FBgn0039358; E-values range from 1.1e-135 to 8.2e-136 for the different isoforms and a sequence identity of 97.6%). *CG5028* is located on the Muller E element with five (D isoform) or six coding exons (A, B, and C isoforms). The next best blastp hit is to the gene *CG6439*, with an E-value of 2.2e-63 and a sequence identity of 68.6%. Hence if *GF22695* were a real protein-coding gene, then it is either a putative ortholog of *CG5028* or a novel paralog derived from *CG5028*.

A tblastn search of the *D. melanogaster CG5028* gene against the *D. ananassae* genome assembly placed the putative ortholog of this gene on scaffold_13340 (corresponding to the *Gnomon* prediction *GF16768*; FlyBase ID: FBgn0093789). The *GF16768* gene prediction consists of multiple coding exons and it is located on a scaffold that has previously been mapped to the Muller E element (Schaeffer *et al.* 2008). By contrast, the second coding exon of *GF22695* shows sequence similarity to five of the coding exons of *CG5028* (Figure SM2C). Collectively, the available evidence suggests that part of the *CG5028* ortholog in *D. ananassae* might have been captured by helitrons and was subsequently integrated into scaffold_13767.

**Comparison of expression profiles of *D. ananassae* and *D. melanogaster* F element genes**

To gather preliminary evidence on whether the increase in repeat density affects the expression levels of *D. ananassae* F element genes, we re-analyzed the *D. ananassae* and *D. melanogaster* RNA-Seq datasets for adult females and adult males that have previously been produced by the modENCODE project (Chen *et al.* 2014). The five number summaries show that the *D. ananassae* and *D. melanogaster* F element genes have similar ranges of Reads Per Kilobase of transcript per Million mapped reads (RPKM) values in the adult females and adult males RNA-Seq samples (Table SM2).

| Metric | Adult Females | | Adult Males | |
| --- | --- | --- | --- | --- |
|  | ***D. ana*: F**  **(modENCODE)** | ***D. mel*: F**  **(modENCODE)** | ***D. ana*: F**  **(modENCODE)** | ***D. mel*: F**  **(modENCODE)** |
| Min. | 1.15 | 0.73 | 1.33 | 2.35 |
| 1^st^ Quartile | 10.85 | 5.84 | 8.38 | 11.19 |
| Median | 23.22 | 22.08 | 26.82 | 21.07 |
| 3^rd^ Quartile | 64.87 | 59.06 | 49.09 | 50.29 |
| Max. | 3759.00 | 10370.00 | 972.40 | 3422.00 |

Table SM2 Five-number summary of the RPKM values (obtained from Chen *et al.* 2014) for the initial coding exon of *D. ananassae* and *D. melanogaster* F element genes in the adult females and adult males RNA-Seq samples.

Similarly, violin plots of RPKM values show that *D. ananassae* F element genes exhibit a similar range of expression levels compared to *D. melanogaster* F element genes in the adult males and adult females RNA-Seq samples (Figure SM3). Wilcoxon rank-sum tests (Mann and Whitney 1947) show that the differences in the distributions of RPKM values in the adult females (p-value = 0.417) and the adult males (p-value = 0.840) samples are not statistically significant. Hence the preliminary comparative expression analysis suggests that *D. ananassae* F element genes have adapted to the high repeat density environment to facilitate their expressions within this heterochromatic domain.

Figure SM3. Violin plots show that the expression levels of *D. ananassae* F element genes are similar to *D. melanogaster* F element genes. The distributions of RPKM expression values for 63 initial coding exons of *D. melanogaster* F element genes and the orthologous coding exons for the *D. ananassae* F element genes were shown in violin plots. A violin plot is composed of a kernel density plot and a boxplot, where the black dot denotes the median and the darker regions demarcate the interquartile range. A pseudocount of 0.0001 was added to the RPKM values in order to display the results in the log_10_ scale. The RPKM values were obtained from Table S5 of Chen *et al.* 2014.

# Supplemental Methods

## General overview

The improved sequences, gene annotations, and the results of the bioinformatic analyses for *D. ananassae* are available through the *D. ananassae* Oct. 2015 (GEP/DanaImproved) assembly at the GEP UCSC Genome Browser mirror ([http://gander.wustl.edu](http://gander.wustl.edu/)). The *D. melanogaster* release 6 assembly was produced by the Berkeley *Drosophila* Genome Project [BDGP; (Hoskins *et al.* 2015)] and the release 6.06 gene annotations for *D. melanogaster* were obtained from the FlyBase (Attrill *et al.* 2016) FTP site at <ftp://ftp.flybase.net/releases/FB2015_03/dmel_r6.06/>. These *D. melanogaster* gene annotations and the results of additional bioinformatic analyses described in this study are available through the *D. melanogaster* Aug. 2014 (BDGP Release 6 + ISO1 MT/dm6) assembly on the GEP UCSC Genome Browser mirror.

The Kent utilities (Kent *et al.* 2002) were used in most of the data conversions and in the construction of the GEP UCSC Genome Browser instances for *D. ananassae* and *D. melanogaster*. *BEDTools* (Quinlan and Hall 2010) was used to identify intersections, unions, and differences among the features. Custom scripts were used to facilitate the data conversions and analyses. Some of the single-threaded tools were run in parallel using *GNU Parallel* (Tange 2011). Most of the analyses were run on a Dell Precision T5400 Linux server (with 8 Xeon processors and 8 GB of RAM) and on a MacBook Pro laptop (with an Intel Core i7 processor and 8 GB of RAM). Some of the more CPU- and memory-intensive tasks (*e.g.*, RNA-Seq analysis, *de novo* repeat finding) were run on a virtual machine with 22 virtual cores and 42 GB of RAM.

## Sequence improvement

The *D. ananassae* CAF1 assembly obtained from the AAA: 12 *Drosophila* Genomes web site (<http://eisenlab.org/AAA/index.html>) contained a *reads.placed* file that specified the placements of fosmid end reads and subclone reads in the *D. ananassae* CAF1 assembly. The selected regions of the *D. ananassae* F and D element scaffolds were partitioned into overlapping fosmids based on this *reads.placed* file. Sanger reads placed in the analysis regions were retrieved from the National Center for Biotechnology Information (NCBI) Trace Archive (<https://www.ncbi.nlm.nih.gov/Traces/trace.cgi>) and then assembled using the *Phred*, *Phrap*, and *Consed* software package (Ewing and Green 1998; Gordon *et al.* 1998).

In collaboration with the staff at the McDonnell Genome Institute at Washington University, misassemblies and gaps in each *D. ananassae* fosmid project were resolved by manual sequence improvement. Additional sequence improvement protocols and tools were developed to address the high rates of misassemblies in the *D. ananassae* F element projects. These additional tools and curriculum materials are available through the “Resolving Misassemblies” section of the GEP website (<http://gep.wustl.edu/>; under Curriculum → Washington University → Finishing and Sequence Improvement). For quality control purposes, each project was completed by at least two GEP students working independently. The final assemblies were reviewed and reconciled by the staff at the McDonnell Genome Institute or by experienced undergraduates working under the supervision of GEP staff to produce the final consensus sequences used in the subsequent analyses.

The fosmid clones for the *D. ananassae* analysis regions improved_13010, improved_13034_2, and improved_13337 were obtained from the *Drosophila* Genomics Resource Center (DGRC) at Indiana University. These fosmids were used as templates for additional sequencing reactions and to produce the restriction digests used to verify the final assemblies. Each fosmid was digested with four restriction enzymes (*Eco*RI, *Eco*RV, *Hind*III, *Sac*I). The fragment sizes of at least two of these real restriction digests must be in congruence with the corresponding *in silico* digests of the final consensus sequence in order to satisfy the sequence improvement standard.

During the course of this study, the *D. ananassae* fosmid library became unavailable from the DGRC. Hence additional sequencing data for the improved_13034_1 region was produced by sequencing genomic PCR products. Projects within the improved_13034_1 region were confirmed by subreads produced by the Pacific Biosciences (PacBio) RS II sequencer (see Materials and Methods; Figure S2B). The PacBio subreads were aligned against the CAF1 assembly using *BLASR* (Chaisson and Tesler 2012) with the parameters -minPctIdentity 80 -bestn 1. A subset of the PacBio subreads that cover the same region as the fosmid project in the CAF1 assembly were selected. These PacBio subreads were aligned against the improved consensus sequence of the fosmid project using *MegaBLAST* (Morgulis *et al.* 2008) with default parameters and an Expect Threshold of 1e-100 in order to verify the final assembly and to provide more accurate gap size estimates. Dot plot alignments between the PacBio subreads and the improved fosmid sequence were produced using *Gepard* (Krumsiek *et al.* 2007) with default parameters.

## Comparison of the CAF1 and improved assembly

Repetitive sequences within the improved *D. ananassae* assembly were soft-masked by *WindowMasker* (Morgulis *et al.* 2006) using default parameters. The masked improved assembly was aligned against the *D. ananassae* CAF1 assembly using discontiguous *MegaBLAST* (Morgulis *et al.* 2008) with the following parameters: -evalue 1e-50 -max_target_seqs 100 -task dc-megablast -db_soft_mask 30. These *MegaBLAST* alignments were filtered and chained together using the UCSC Chain and Net alignment protocol (Kent *et al.* 2003). The Net alignments were then converted into PSL and BED formats to facilitate the comparisons between the two assemblies.

## Gene annotations

GEP students used a mirror of the UCSC Genome Browser (Kent *et al.* 2002) to examine different lines of experimental (*e.g.*, RNA-Seq) and computational evidence (*e.g.*, sequence similarity to *D. melanogaster* protein sequences, gene predictions, splice junction predictions) to construct the *D. ananassae* gene models. The paired-end *D. ananassae* RNA-Seq data from adult females and adult males used in the gene annotation process were produced by the modENCODE project (Graveley *et al.* 2011). The RNA-Seq data were obtained from the European Nucleotide Archive (ENA) at the European Molecular Biology Laboratory - European Bioinformatics Institute (EMBL-EBI) using the NCBI Sequence Read Archive (SRA) accession number SRP006203. The evidence tracks derived from these RNA-Seq datasets included RNA-Seq read coverage, splice junction predictions from *TopHat2* (Kim *et al.* 2013), and assembled transcripts from *Cufflinks* (Trapnell *et al.* 2010) and *Oases* (Schulz *et al.* 2012).

Each annotation project was completed by at least two GEP students working independently. Experienced students working under the supervision of GEP staff reconciled these gene annotations using *Apollo* (Lewis *et al.* 2002) to create the gene models analyzed in this study. The Annotation Instruction Sheet (available on the GEP web site at [http://gep.wustl.edu](http://gep.wustl.edu/)) describes the protocols for handling cases where the annotations were ambiguous because of insufficient evidence. Additional curricula (*e.g.*, walkthroughs, presentations) that describe the GEP annotation protocol are available on the GEP web site.

## Identification of additional *D. ananassae* F element scaffolds

Conceptual translations of the coding exons of *D. melanogaster* genes were aligned against the improved *D. ananassae* assembly using the *tblastn* program in WU BLAST (Gish 1996) with the following parameters: -e=1e-2 -topComboN=1 -links -hspsepSmax=10000 -hspsepQmax=1000 -matrix=PAM40 -Q=7 -R=2 -xmlcompact. The *tblastn* results were converted into PSL format using *blastXmlToPsl* in the Kent utilities. Suboptimal matches were retained if their alignment scores were within 10% of the score of the best match, irrespective of their placements in the *D. ananassae* assembly. Suboptimal matches were also kept if they were located within 10kb of the best match and had an alignment score of at least 20.

These matches to the coding exons of each *D. melanogaster* gene were used to define the approximate placement of the *D. melanogaster* ortholog in the improved *D. ananassae* assembly. Alignments to coding exons of the same *D. melanogaster* gene that were located within 100 kb of each other in the *D. ananassae* assembly were grouped together into a single region. Extra paddings of 30 kb were added to the start and end of each region. All isoforms of the *D. melanogaster* gene were then compared against this *D. ananassae* region using *SPALN2* (Iwata and Gotoh 2012) with the following parameters: -pw -O1 -Tdromel -yS -yX.

The *SPALN2* protein alignments and the *tblastn* coding sequence alignments were added to the improved *D. ananassae* assembly on the GEP UCSC Genome Browser mirror. Regions within the *D. ananassae* assembly that showed significant sequence similarity to *D. melanogaster* F element genes were analyzed to produce the list of putative *D. ananassae* F element scaffolds.

## *Tallymer* repeat analysis

In order to use *Tallymer* (Kurtz *et al.* 2008) to estimate the total repeat density, the *oocratio* subprogram was used to identify the word size (k) where 95% of the words were unique in the *D. melanogaster* and *D. ananassae* assemblies. Using a word size of 17 (k=17), 94.7% of the words were unique in the *D. melanogaster* assembly. By contrast, only 91.1% of the words were unique with k=17 in the *D. ananassae* assembly. Using k=19 increased the percentage of unique words in the *D. ananassae* assembly to 93.1%. Subsequent increases in word sizes resulted in only minor increases in the percentage of unique words (*e.g.*, 93.8% of the words were unique at k=30). Hence, k=19 was used in the *Tallymer* analysis of the *D. ananassae* assembly.

The *mkindex* subprogram in *Tallymer* was used to construct the list of k-mers that appeared at least four times (-minocc 4) in each assembly. These *D. melanogaster* and *D. ananassae* *Tallymer* indices were used with the *search* subprogram to locate instances of each k-mer in the *D. melanogaster* and *D. ananassae* assemblies. Overlapping k-mer matches were merged into a single interval and repeats shorter than 40 bp were filtered from the *Tallymer* search results.

## Determination of the cutoff scores for *Wolbachia* searches

To determine the cutoff scores for the alignments between the three *Wolbachia* assemblies and the *Drosophila* assemblies, the *D. melanogaster* and *D. ananassae* genome assemblies were shuffled using *esl-shuffle* (--seed 1000) in the *Easel* library [part of the distribution for *HMMER3*; (Eddy 2011)], while maintaining the dinucleotide frequencies of the original assembly (-d). These shuffled assemblies were searched against the *wAna*, *wRi*, and *wMel* assemblies using *RepeatMasker* with the same parameters as the original searches (-e wublast -s -nolow). The 95^th^ percentile of the Smith-Waterman scores (SW scores) from the search against the shuffled assembly was used as the cutoff score. The cutoff scores for the three *Wolbachia* assemblies ranged from 255.6–263.4 for *D. melanogaster* and ranged from 250.7–256.0 for *D. ananassae*. *RepeatMasker* matches in the *D. melanogaster* and improved *D. ananassae* assemblies with a SW score below these cutoff scores were removed from the search results prior to the *Wolbachia* density analysis.

These shuffled assemblies were also used to determine the cutoff scores for the *CENSOR* (Kohany *et al.* 2006) alignments of *Wolbachia* protein sequences against the *D. melanogaster* and *D. ananassae* assemblies. The 95^th^ percentile of the *CENSOR* alignment scores for the shuffled assemblies were used as the cutoff scores and they ranged from 112.0–116.2 in *D. melanogaster* and ranged from 94.8–106.2 in *D. ananassae* for the three *Wolbachia* species. *CENSOR* matches with scores below these cutoff scores were filtered from the results.

## Determination of the *D. ananassae* scaffold coverage in the *wAna* assembly

The regions of the *wAna* assembly that aligned to the improved *D. ananassae* assembly were extracted from the *RepeatMasker* .out file produced as part of the “Estimating the density of *Wolbachia* fragments” analysis (see Materials and Methods). The *wAna* coordinates were converted into BED format and the alignment coverage of *D. ananassae* scaffolds in the *wAna* assembly was determined by the *genomecov* subprogram in *BEDTools*. The BedGraph output was converted into BigWig format to show the *D. ananassae* scaffold coverage in the *Wolbachia wAna* January 2005 (TIGR) assembly on the GEP UCSC Genome Browser mirror.

## Construct the reference gene set for codon bias analyses

The coding DNA sequences of the *D. melanogaster* gene models (release 6.06) and the *D. ananassae Gnomon* gene predictions (release 1.04) were obtained from the FlyBase FTP site at <ftp://ftp.flybase.net/releases/FB2015_03/>. These coding sequences were analyzed using the *scnRCA* (O’Neill *et al.* 2013) program (with the parameters: -i r -g true -d 2.0 -p 1.0 -m -1) to construct the reference gene sets for the Codon Adaptation Index (CAI) analysis. The codon frequency of the reference gene set was determined using the *cusp* program in the *EMBOSS* package (Rice *et al.* 2000). The resulting file was used with the *cai* program (via the -cfile parameter) to calculate the CAI value for each gene in the analysis regions.

## Mapping of ChIP-Seq reads

The paired-end ChIP-Seq reads for H3K4me2, H3K9me2, H3K27me3, and input DNA were mapped against the improved *D. ananassae* assembly using *BWA-MEM* (Li 2013) with default parameters. The mate coordinates were added to the alignments and the alignments were converted into BAM format using the *fixmate* subprogram in *SAMtools* (Li *et al.* 2009). The BAM files were then sorted by coordinates using the *sort* subprogram in *SAMtools* and duplicate reads were marked using the *MarkDuplicates* subprogram in *Picard* (available at <http://broadinstitute.github.io/picard>). Each BAM file was analyzed using the *CollectInsertSizeMetrics* subprogram in *Picard* to estimate the size of the DNA fragments between the Illumina adapters (i.e., insert size) and the median absolute deviation (MAD) of the insert size. This analysis produced an estimated median insert size of 183 bp and a MAD of 41 bp for the entire set of ChIP and input samples.

Because the read lengths for the *D. melanogaster* ChIP-Seq samples were shorter than 70bp, the *BWA-backtrack* algorithm [*aln/samse*; (Li and Durbin 2009)] was used with default parameters to map the ChIP-Seq reads against the *D. melanogaster* assembly. The alignments were converted into BAM format and sorted by coordinates using the *sort* subprogram in *SAMtools*. Duplicate reads were identified using the *MarkDuplicates* subprogram in *Picard*. Because the ChIP-Seq reads were unpaired, the *CollectInsertSizeMetrics* subprogram in *Picard* could not be used to estimate the insert size and the MAD for the insert size.

## Estimate the effective genome sizes

Simulated reads were produced using *ART* (Huang *et al.* 2012) that resulted in 10-fold genome coverage. The simulated reads were mapped against the *D. melanogaster* and *D. ananassae* genomes using the same alignment tool and parameters as described in the “Mapping of ChIP-Seq reads” section. The BAM alignment files were analyzed using the *genomecov* subprogram in *BEDTools* to determine the alignment coverage*.* The effective genome size corresponded to the number of bases within each assembly that had at least one mapped simulated read.

For *D. ananassae*, the insert size (--len) and the standard deviation (--sdev) used to generate the simulated reads were determined by the median insert size (183 bp) and the median MAD (41 bp) for the entire set of ChIP and input samples. A read length of 100 instead of 101 was used to generate the simulated reads based on the built-in profiles for HiSeq 2000 that were available in *ART*. The simulated reads (with 10x genome coverage) were produced using the following parameters: --fcov 10 --noALN --paired --rndSeed 4135 --seqSys HS20 --len 100 --mflen 183 --sdev 41. Mapping and coverage analyses of the *D. ananassae* simulated reads resulted in 92.3% genome coverage and an effective genome size estimate of 2.1e8 bp.

The simulated reads for *D. melanogaster* were produced using *ART* with the following parameters: --fcov 10 --noALN --rndSeed 4135 --seqSys GA2 --len 50. Mapping and coverage analyses of the *D. melanogaster* simulated reads resulted in 99.2% genome coverage and an effective genome size estimate of 1.4e8 bp.

## ChIP-Seq peak calling

The ChIP-Seq alignments for H3K4me2 were analyzed using the *callpeak* subprogram in *MACS2* (Zhang *et al.* 2008) with the default minimum false discovery rate (FDR) cutoff (--qvalue) of 0.01. The signal profiles were analyzed to identify subpeaks within the enriched regions (--call-summits). The *callpeak* subprogram was run in broad peak calling mode (--broad) for the H3K9me2 and H3K27me3 samples with the default qvalue cutoff (--broad-cutoff) of 0.1. For both the regular and broad peak calling, the parameters specified above were used in conjunction with the effective genome size (--gsize; 2.1e8 for *D. ananassae* and 1.4e8 for *D. melanogaster*), and the following parameters: --buffer-size 1000 --SPMR -B.

The *bdgcmp* subprogram in *MACS2* (with the parameters -m logLR -p 0.00001) was used to calculate the log likelihood enrichment ratios (LLR) of the ChIP samples compared to DNA input controls. Because some of the intervals in the bedGraph file produced by *bdgcmp* extended beyond the size of the chromosome, the extra regions in the bedGraph file were removed using the *slop* subprogram in *BEDTools* with no additional bases added to each interval (-b 0) and then clipped with the *bedClip* tool (part of the Kent utilities). The clipped bedGraph files were converted into BigWig format using *bedGraphToBigWig* and the correlations between biological replicates were determined by the *wigCorrelate* tool in the Kent utilities. For *D. melanogaster*, the correlations between biological replicates were 0.582 for H3K9me2, 0.976 for H3K4me2, and 0.799 for H3K27me3. For *D. ananassae*, the correlations between the biological replicates were 0.940 for H3K9me2, 0.977 for H3K4me2, and 0.906 for H3K27me3.

Because the *wigCorrelate* results showed that the LLR of the biological replicates were generally highly correlated with each other, the BAM alignments from the two replicates were merged and then analyzed with *MACS2*. The H3K4me2 sample was analyzed using the regular peak calling mode while the H3K9me2 and H3K27me3 samples were analyzed using the broad peak calling mode with the same parameters as specified above. The construction of the shifting model was skipped (--nomodel), and the same extension size (--extsize 73) and shift size (--shiftsize 37) were used for all samples. In addition, the genome size (--gsize) of 2.1e8 and the bandwidth (--bw) of 183 were used for the *D. ananassae* samples, while a genome size of 1.4e8 and a bandwidth of 300 were used for the *D. melanogaster* samples.

The LLR evidence tracks for the merged samples were created using the *bdgcmp* subprogram in *MACS2* with the same analysis procedure as the individual replicates (described above). The LLR metagene profiles were created using the technique described in the “Melting temperature metagene profile” section of Materials and Methods.

## Calculate RNA-Seq read count for each *D. ananassae* gene

The *D. ananassae* paired-end RNA-Seq reads were trimmed by quality using the *pe* subprogram in *Sickle* (available at <https://github.com/najoshi/sickle>) with default parameters and the Sanger quality type (--qual-type sanger). The trimmed RNA-Seq reads from all seven samples were mapped against the improved *D. ananassae* assembly using two rounds of *HISAT2* (Kim *et al.* 2015). The first round of the *HISAT2* analysis was used to identify novel splice junctions (--novel-splicesite-outfile) and it was run with the following parameters: --min-intronlen 30 --max-intronlen 150000 --dta-cufflinks. The second round of the *HISAT2* analysis used the splice junctions identified in the first round (--novel-splicesite-infile) with the same read mapping parameters as the first round to improve the alignment sensitivity.

The *D. ananassae* (release 1.04) gene annotations were obtained from the FlyBase FTP site (<ftp://ftp.flybase.net/releases/FB2015_03/dana_r1.04/gtf/>). These gene annotations were lifted from the *D. ananassae* CAF1 assembly to the improved assembly using the *liftOver* program in the Kent utilities. The RNA-Seq read counts for each gene were determined by analyzing the BAM alignment files for the seven samples with *htseq-count* (Anders *et al.* 2015) using default parameters. The read count tables for the samples with multiple replicates (i.e., adult females, adult males, embryos) were merged.

## Correlation between CAI and gene expression levels

To ascertain the relationships between CAI and rlog expression levels among all *D. ananassae* genes, release 1.04 of the *D. ananassae Gnomon* gene annotations were used in both the CAI and the rlog analyses. The CAI for the isoform with the largest total coding exon size (*i.e.*, the most comprehensive isoform) of each *D. ananassae Gnomon* gene prediction was determined using the approach described in the “Construct the reference gene set for codon bias analyses” section of Supplemental Methods. The rlog expression levels of each *Gnomon* each prediction has previously been determined using the approach described in the “RNA-Seq expression analysis” section of Materials and Methods.

The Spearman’s rank correlation coefficient between CAI and rlog were determined using the *corr* function in *R* (with method=“spearman”). The CAI versus rlog scatterplots and the LOESS regression lines for the *D. ananassae* F element and the base of the D element were produced using the same approach as described under the “Codon bias analysis” section of Materials and Methods. The *smoothScatter* function in *R* was used to produce the smoothed color density representation of the CAI versus rlog scatterplots for the Gnomon gene predictions on all scaffolds. The smoothing parameters for the LOESS regression lines were determined by the *loess.as* function in the *R* package fANCOVA with the following parameters: degree=1, criterion=“gcv”, family=“symmetric”. The LOESS regression lines were added to the smoothed scatterplot using the *loess.smooth* function in R, with the parameters estimated by *loess.as* and evaluation=50.

## Comparative expression analysis of *D. ananassae* and *D. melanogaster* F element genes

As part of a comparative analysis of the *Drosophila* transcriptome, Chen and colleagues compared the expression levels of the initial coding exons of orthologous genes in 15 *Drosophila* species (Chen *et al.* 2014). To compare the expression levels of *D. ananassae* and *D. melanogaster* F element genes, we extracted the Reads Per Kilobase of transcript per Million mapped reads (RPKM) values for the *D. ananassae* and *D. melanogaster* F element genes in the adult males and adult females RNA-Seq samples (from Table S5 of Chen *et al.* 2014), and compared the RPKM distributions. Coding exons with either no RPKM values (NA) or with less than 95% exon coverage (LC) in Table S5 were omitted from this analysis. The violin plots were produced by a modified version of the *vioplot* function in *R*, as described in the “Gene characteristics analysis” section of Materials and Methods. The Wilcoxon rank-sum tests were performed using the *wilcox.test* in *R*, and a Type I error (α) of less than or equal to 0.05 were considered to be statistically significant.

## Constructing the centroid repeat library

**Centroid repeat library overview**: The centroid transposon library was produced by augmenting the *Drosophila* transposon sequences in the *RepBase* library (release 20150807) with *Drosophila* helentrons (Thomas *et al.* 2014) and *D. ananassae* transposons identified using five *de novo* repeat finders. The protocols for constructing the *de novo* repeat libraries were based on the *REPET* *TEdenovo* pipeline (Flutre *et al.* 2011). The following sections described the protocols used to construct and classify each *de novo* repeat library, and the protocols for combining sequences from these repeat libraries to create the final centroid repeat library.

***Drosophila* transposons in RepBase**: The *RepeatMasker* (Smit *et al.* 2013) version of the RepBase repeat library [release 20150807; (Jurka *et al.* 2005)] was obtained from the Genetic Information Research Institute (GIRI) web site at <http://www.girinst.org/>. *Drosophila* transposon sequences were extracted from the RepBase library using the *queryRepeatDatabase.pl* script in the *RepeatMasker* package. Repeats that were classified as ARTEFACT, Low_complexity, RNA, Simple_repeat, Satellite, and Other were removed from the *Drosophila* transposon library.

***Drosophila* Helentron sequences**: The GenBank accession numbers and the coordinates for the *Drosophila* *ananassae*, *D. willistoni*, and *D. yakuba* helentrons and Helentron associated INterspersed Elements (HINE) were obtained from Additional file 8 of the manuscript by Thomas and colleagues (Thomas *et al.* 2014). The sequence for each of these repeats was obtained from the corresponding records at the NCBI Nucleotide database.

***ReAS***: The *Drosophila* 12 Genomes Consortium (Drosophila 12 Genomes Consortium *et al.* 2007) has constructed a repeat library based on the *ReAS* (Li *et al.* 2005) analysis of the Sanger sequencing reads that were produced as part of the *D. ananassae* Comparative Analysis Freeze 1 (CAF1) assembly. The *D. ananassae* *ReAS* transposon library was obtained from FlyBase (<ftp://ftp.flybase.net/genomes/aaa/transposable_elements/ReAS/v2/consensus_fasta/>).

***LTRHarvest***: Putative LTR retrotransposons in the improved *D. ananassae* assembly were identified using *LTRHarvest* (Ellinghaus *et al.* 2008) with the following parameters: -seed 76 -minlenltr 116 -maxlenltr 800 -mindistltr 2280 -maxdistltr 8773 -similar 91 -xdrop 7 -mat 2 -mis -2 -ins -3 -del -3 -mintsd 4 -maxtsd 20 -vic 60 -overlaps best -longoutput. Internal features within the LTR retrotransposons were identified using *LTRdigest* (Steinbiss *et al.* 2009). The conserved domains associated with retrotransposons used in the *LTRdigest* analysis were obtained by searching release 27.0 of the *Pfam* database (Finn *et al.* 2016) using the following keywords: retrotransposon, *env* transposon, reverse transcriptase, retroelements, and *gag* transposon. The *D. ananassae* tRNA sequences (release 1.04) were obtained from the FlyBase FTP site at <ftp://flybase.net/genomes/Drosophila_ananassae/dana_r1.04_FB2015_01/fasta/>. In conjunction with the conserved domains (-hmms) and tRNA sequences (-trnas), *LTRdigest* was run with the following parameters: -pptradius 30 -pptlen 8 30 -pptrprob 0.97 -uboxlen 3 30 -pptuprob 0.91 -pbsradius 30 -pbsalilen 11 30 -pbsoffset 0 5 -pbstrnaoffset 0 40 -pbsmaxedist 1 -pbsmatchscore 5 -pbsmismatchscore -10 -pbsinsertionscore -20 -pbsdeletionscore -20 -pdomevalcutoff 1e-6 -maxgaplen 50.

The predictions from *LTRHarvest* were filtered and clustered into families based on the protocol developed by Steinbiss and colleagues (Steinbiss *et al.* 2012). The *LTRHarvest* predictions that contained matches to protein domains associated with LTR retrotransposons were identified using the *select* subprogram in *GenomeTools* (Gremme *et al.* 2013). These predictions were clustered into families using the *ltrclustering* subprogram in *GenomeTools* with the following parameters: -psmall 80 -plarge 30. LTR families with less than three members were filtered and the centroid sequences for the remaining LTR families were identified using the *UCLUST* algorithm in *USEARCH* (Edgar 2010) with the following parameters: -cluster_fast -id 0.8 -centroids. These centroid sequences were classified using *PASTEC* (Hoede *et al.* 2014) and only the subset of repeats that were classified as LTR retrotransposons was kept.

***RepeatModeler***: The improved *D. ananassae* assembly was analyzed using *RepeatModeler* (Smit and Hubley 2008) with the *WU BLAST* (Gish 1996) search engine and default parameters. The original repeat classifications from *RepeatModeler* were removed and the repeats in the library were re-classified using *PASTEC* (Hoede *et al.* 2014).

***Tedna***: The paired-end Illumina genomic reads from the whole genome sequencing of the *Wolbachia*-cured *D. ananassae* (stock number 14024-0371.13) were obtained from the European Nucleotide Archive (ENA) at the European Molecular Biology Laboratory - European Bioinformatics Institute (EMBL-EBI) using the accession number SRR491673. The paired-end Illumina reads produced by the Illumina Genome Analyzer IIx sequencer had an estimated insert size of 300 bp.

In order to estimate the optimal transposon assembly parameters for *Tedna* (Zytnicki *et al.* 2014), multiple thresholds (-t = 30, 40, and 50) and k-mer sizes (-k = 41, 51, 61, and 71) were tested with the insert size of 300 bp (-i 300). The *Tedna* repeat libraries constructed with each combination of threshold and k-mer size were used with *RepeatMasker* (Smit *et al.* 2013) to identify repeats in the *D. ananassae* assembly. *RepeatMasker* was run at the most sensitive setting (-s) with the WU BLAST search engine (-e wublast) and without masking low complexity and simple repeats (-nolow). The threshold of 50 and the k-mer size of 41 were used to construct the final *Tedna* library because these parameters produced a repeat library that resulted in the highest repeat density estimate (31.3%) of the *D. ananassae* assembly among the parameters tested.

***dnaPipeTE***: The paired-end Illumina genomic reads used in the *Tedna* analysis (i.e. SRR491673) were also analyzed using the *dnaPipeTE* (Goubert *et al.* 2015) transposon assembler. Illumina reads that were at least 70 bp long were selected using the *reformat.sh* script in *BBMap* (Bushnell B., <https://sourceforge.net/projects/bbmap/>). In order to optimize the sample size for the transposon assembly, *dnaPipeTE* was run using multiple sample sizes (-sample_size = 300000, 500000, 800000, and 1000000) with four iterations (-sample_number 4). The quality of each assembly was measured by the N25 length (i.e. the length of the contig this size and greater that accounted for 25% of the total size of the repeat library). The sample size of 800,000 was used to construct the final *dnaPipeTE* library because it resulted in the highest N25 (1745 bp) among the four sample sizes tested.

**Repeat classification**: Repeat sequences in each *de novo* repeat library were classified using *PASTEC* (Hoede *et al.* 2014). As part of the configuration of *PASTEC*, the *REPET* edition of the RepBase library (release 20.05) was obtained from the GIRI web site (<http://www.girinst.org/>). The Hidden Markov Model (HMM) profile bank for *REPET* (i.e. ProfilesBankForREPET_Pfam26.0_GypsyDB.hmm.tar.gz) was obtained from the *REPET* web site (<https://urgi.versailles.inra.fr/download/repet/>). The *D. melanogaster* ribosomal RNA (rRNA) sequences (release 6.06) were obtained from the FlyBase FTP site (<ftp://flybase.net/genomes/Drosophila_melanogaster/dmel_r6.06_FB2015_03/fasta/>).

The *PASTEClassifier.py* script in the *PASTEC* package was run on each unclassified *de novo* repeat library (i.e. *ReAS*, *RepeatModeler*, *Tedna*, and *dnaPipeTE*) using default parameters. The output sequences were renamed (-w) according to the classification system developed by Wicker and colleagues (Wicker *et al.* 2007). The reverse complement (-r) of the repeat sequence was reported if *PASTEC* determined (based on the distribution of transposon features) that the repeat was on the minus strand.

**Combine transposon sequences from multiple repeat libraries**: Each classified *de novo* repeat library was used with *RepeatMasker* in a search against the improved *D. ananassae* genome assembly with the following parameters: -nolow -s -e wublast. Unclassified repeats (noCat) were retained in the repeat library only if there were at least five matches that accounted for more than 1kb of the improved *D. ananassae* assembly. Repeats that were classified by *PASTEC* as PotentialChimeric, rDNA, and PotentialHostGene were also removed from the repeat library.

Sequences from all of the filtered and classified *de novo* repeat libraries were combined and then partitioned based on completeness. Incomplete repeats were clustered using *cd-hit-est* in the *CD-HIT* (Li and Godzik 2006) package with the following parameters: -c 0.80 -n 5 -d 0.

Sequences in the *Drosophila* helentrons and HINE library, the *Drosophila* RepBase library, the complete *de novo* repeats, the LTR retrotransposons identified using *LTRHarvest*, and the clustered incomplete *de novo* repeats were each sorted by size in descending order and then combined into a single file. These sequences were clustered using the *cluster_fast* command in the *USEARCH* program (Edgar 2010) with the identity threshold of 0.8 (-id 0.8) in order to create the cluster centroids sequences (-centroids) in the centroid repeat library.

Using this centroid repeat library, *RepeatMasker* was run against the improved *D. ananassae* assembly with the following parameters: -nolow -s -e wublast. Overlapping repeats were merged only if they belonged to the same repeat class. If the repeats that overlapped with each other belonged to different repeat classes, then the region of overlap was reclassified as “Overlapping” in the transposon density analysis.

# Literature cited

Anders, S., P. T. Pyl, and W. Huber, 2015 HTSeq — a Python framework to work with high-throughput sequencing data. Bioinforma. Oxf. Engl. 31: 166–169.

Angov, E., 2011 Codon usage: nature’s roadmap to expression and folding of proteins. Biotechnol. J. 6: 650–659.

Attrill, H., K. Falls, J. L. Goodman, G. H. Millburn, G. Antonazzo *et al.*, 2016 FlyBase: establishing a Gene Group resource for *Drosophila melanogaster*. Nucleic Acids Res. 44: D786-792.

Chaisson, M. J., and G. Tesler, 2012 Mapping single molecule sequencing reads using basic local alignment with successive refinement (BLASR): application and theory. BMC Bioinformatics 13: 238.

Chen, Z.-X., D. Sturgill, J. Qu, H. Jiang, S. Park *et al.*, 2014 Comparative validation of the *D.* *melanogaster* modENCODE transcriptome annotation. Genome Res. 24: 1209–1223.

Cleveland, W. S., and S. J. Devlin, 1988 Locally Weighted Regression: An Approach to Regression Analysis by Local Fitting. J. Am. Stat. Assoc. 83: 596.

*Drosophila* 12 Genomes Consortium, A. G. Clark, M. B. Eisen, D. R. Smith, C. M. Bergman *et al.*, 2007 Evolution of genes and genomes on the *Drosophila* phylogeny. Nature 450: 203–218.

Eddy, S. R., 2011 Accelerated Profile HMM Searches. PLoS Comput. Biol. 7: e1002195.

Edgar, R. C., 2010 Search and clustering orders of magnitude faster than BLAST. Bioinforma. Oxf. Engl. 26: 2460–2461.

Ellinghaus, D., S. Kurtz, and U. Willhoeft, 2008 LTRharvest, an efficient and flexible software for *de novo* detection of LTR retrotransposons. BMC Bioinformatics 9: 18.

Ewing, B., and P. Green, 1998 Base-calling of automated sequencer traces using phred. II. Error probabilities. Genome Res. 8: 186–194.

Finn, R. D., P. Coggill, R. Y. Eberhardt, S. R. Eddy, J. Mistry *et al.*, 2016 The Pfam protein families database: towards a more sustainable future. Nucleic Acids Res. 44: D279-285.

Flutre, T., E. Duprat, C. Feuillet, and H. Quesneville, 2011 Considering transposable element diversification in *de novo* annotation approaches. PloS One 6: e16526.

Gish, W., 1996 WU BLAST. Retrieved from <http://blast.wustl.edu/>

Gordon, D., C. Abajian, and P. Green, 1998 Consed: a graphical tool for sequence finishing. Genome Res. 8: 195–202.

Goubert, C., L. Modolo, C. Vieira, C. ValienteMoro, P. Mavingui *et al.*, 2015 *De novo* assembly and annotation of the Asian tiger mosquito (*Aedes albopictus*) repeatome with dnaPipeTE from raw genomic reads and comparative analysis with the yellow fever mosquito (*Aedes aegypti*). Genome Biol. Evol. 7: 1192–1205.

Graveley, B. R., A. N. Brooks, J. W. Carlson, M. O. Duff, J. M. Landolin *et al.*, 2011 The developmental transcriptome of *Drosophila melanogaster*. Nature 471: 473–479.

Gremme, G., S. Steinbiss, and S. Kurtz, 2013 GenomeTools: a comprehensive software library for efficient processing of structured genome annotations. IEEEACM Trans. Comput. Biol. Bioinforma. IEEE ACM 10: 645–656.

Hoede, C., S. Arnoux, M. Moisset, T. Chaumier, O. Inizan *et al.*, 2014 PASTEC: an automatic transposable element classification tool. PloS One 9: e91929.

Hoskins, R. A., J. W. Carlson, K. H. Wan, S. Park, I. Mendez *et al.*, 2015 The Release 6 reference sequence of the *Drosophila melanogaster* genome. Genome Res. 25: 445–458.

Huang, W., L. Li, J. R. Myers, and G. T. Marth, 2012 ART: a next-generation sequencing read simulator. Bioinforma. Oxf. Engl. 28: 593–594.

Iwata, H., and O. Gotoh, 2012 Benchmarking spliced alignment programs including Spaln2, an extended version of Spaln that incorporates additional species-specific features. Nucleic Acids Res. 40: e161.

Jurka, J., V. V. Kapitonov, A. Pavlicek, P. Klonowski, O. Kohany *et al.*, 2005 Repbase Update, a database of eukaryotic repetitive elements. Cytogenet. Genome Res. 110: 462–467.

Kent, W. J., R. Baertsch, A. Hinrichs, W. Miller, and D. Haussler, 2003 Evolution’s cauldron: duplication, deletion, and rearrangement in the mouse and human genomes. Proc. Natl. Acad. Sci. U. S. A. 100: 11484–11489.

Kent, W. J., C. W. Sugnet, T. S. Furey, K. M. Roskin, T. H. Pringle *et al.*, 2002 The human genome browser at UCSC. Genome Res. 12: 996–1006.

Kim, D., B. Langmead, and S. L. Salzberg, 2015 HISAT: a fast spliced aligner with low memory requirements. Nat. Methods 12: 357–360.

Kim, D., G. Pertea, C. Trapnell, H. Pimentel, R. Kelley *et al.*, 2013 TopHat2: accurate alignment of transcriptomes in the presence of insertions, deletions and gene fusions. Genome Biol. 14: R36.

Kohany, O., A. J. Gentles, L. Hankus, and J. Jurka, 2006 Annotation, submission and screening of repetitive elements in Repbase: RepbaseSubmitter and Censor. BMC Bioinformatics 7: 474.

Krumsiek, J., R. Arnold, and T. Rattei, 2007 Gepard: a rapid and sensitive tool for creating dotplots on genome scale. Bioinforma. Oxf. Engl. 23: 1026–1028.

Kurtz, S., A. Narechania, J. C. Stein, and D. Ware, 2008 A new method to compute K-mer frequencies and its application to annotate large repetitive plant genomes. BMC Genomics 9: 517.

Lewis, S. E., S. M. J. Searle, N. Harris, M. Gibson, V. Lyer *et al.*, 2002 Apollo: a sequence annotation editor. Genome Biol. 3: RESEARCH0082.

Li, H., 2013 Aligning sequence reads, clone sequences and assembly contigs with BWA-MEM. arXiv arXiv:1303.3997v2:

Li, H., and R. Durbin, 2009 Fast and accurate short read alignment with Burrows-Wheeler transform. Bioinforma. Oxf. Engl. 25: 1754–1760.

Li, W., and A. Godzik, 2006 Cd-hit: a fast program for clustering and comparing large sets of protein or nucleotide sequences. Bioinforma. Oxf. Engl. 22: 1658–1659.

Li, H., B. Handsaker, A. Wysoker, T. Fennell, J. Ruan *et al.*, 2009 The Sequence Alignment/Map format and SAMtools. Bioinforma. Oxf. Engl. 25: 2078–2079.

Li, R., J. Ye, S. Li, J. Wang, Y. Han *et al.*, 2005 ReAS: Recovery of ancestral sequences for transposable elements from the unassembled reads of a whole genome shotgun. PLoS Comput. Biol. 1: e43.

Mann, H. B., and D. R. Whitney, 1947 On a Test of Whether one of Two Random Variables is Stochastically Larger than the Other. Ann. Math. Stat. 18: 50–60.

Morgulis, A., G. Coulouris, Y. Raytselis, T. L. Madden, R. Agarwala *et al.*, 2008 Database indexing for production MegaBLAST searches. Bioinforma. Oxf. Engl. 24: 1757–1764.

Morgulis, A., E. M. Gertz, A. A. Schäffer, and R. Agarwala, 2006 WindowMasker: window-based masker for sequenced genomes. Bioinforma. Oxf. Engl. 22: 134–141.

Moriyama, E. N., and J. R. Powell, 1997 Codon usage bias and tRNA abundance in *Drosophila*. J. Mol. Evol. 45: 514–523.

O’Neill, P. K., M. Or, and I. Erill, 2013 scnRCA: A Novel Method to Detect Consistent Patterns of Translational Selection in Mutationally-Biased Genomes. PloS One 8: e76177.

Quinlan, A. R., and I. M. Hall, 2010 BEDTools: a flexible suite of utilities for comparing genomic features. Bioinforma. Oxf. Engl. 26: 841–842.

Rice, P., I. Longden, and A. Bleasby, 2000 EMBOSS: the European Molecular Biology Open Software Suite. Trends Genet. TIG 16: 276–277.

Schaeffer, S. W., A. Bhutkar, B. F. McAllister, M. Matsuda, L. M. Matzkin *et al.*, 2008 Polytene chromosomal maps of 11 *Drosophila* species: the order of genomic scaffolds inferred from genetic and physical maps. Genetics 179: 1601–1655.

Schulz, M. H., D. R. Zerbino, M. Vingron, and E. Birney, 2012 Oases: robust *de novo* RNA-seq assembly across the dynamic range of expression levels. Bioinforma. Oxf. Engl. 28: 1086–1092.

Smit, A. F. A., and R. Hubley, 2008 RepeatModeler Open-1.0. Retrieved from <http://www.repeatmasker.org/>

Smit, A. F. A., R. Hubley, and P. Green, 2013 RepeatMasker Open-4.0. Retrieved from <http://www.repeatmasker.org/>

Steinbiss, S., S. Kastens, and S. Kurtz, 2012 LTRsift: a graphical user interface for semi-automatic classification and postprocessing of *de novo* detected LTR retrotransposons. Mob. DNA 3: 18.

Steinbiss, S., U. Willhoeft, G. Gremme, and S. Kurtz, 2009 Fine-grained annotation and classification of *de novo* predicted LTR retrotransposons. Nucleic Acids Res. 37: 7002–7013.

Tange, O., 2011 GNU Parallel: The Command-Line Power Tool. Login USENIX Mag. 36: 42–47.

Thomas, J., K. Vadnagara, and E. J. Pritham, 2014 DINE-1, the highest copy number repeats in *Drosophila melanogaster* are non-autonomous endonuclease-encoding rolling-circle transposable elements (Helentrons). Mob. DNA 5: 18.

Trapnell, C., B. A. Williams, G. Pertea, A. Mortazavi, G. Kwan *et al.*, 2010 Transcript assembly and quantification by RNA-Seq reveals unannotated transcripts and isoform switching during cell differentiation. Nat. Biotechnol. 28: 511–515.

Wicker, T., F. Sabot, A. Hua-Van, J. L. Bennetzen, P. Capy *et al.*, 2007 A unified classification system for eukaryotic transposable elements. Nat. Rev. Genet. 8: 973–982.

Zhang, Y., T. Liu, C. A. Meyer, J. Eeckhoute, D. S. Johnson *et al.*, 2008 Model-based analysis of ChIP-Seq (MACS). Genome Biol. 9: R137.

Zytnicki, M., E. Akhunov, and H. Quesneville, 2014 Tedna: a transposable element *de novo* assembler. Bioinforma. Oxf. Engl. 30: 2656–2658.
